# Photochemically driven solid electrolyte interphase for extremely fast-charging lithium-ion batteries

**DOI:** 10.1038/s41467-021-27095-w

**Published:** 2021-11-23

**Authors:** Minsung Baek, Jinyoung Kim, Jaegyu Jin, Jang Wook Choi

**Affiliations:** 1 School of Chemical and Biological Engineering and Institute of Chemical Processes, Seoul National University, Seoul, Republic of Korea; 2 Institute of Battery Technology, SK on, Daejeon, Republic of Korea; 3 Department of Materials Science and Engineering, Seoul National University, Seoul, Republic of Korea

**Keywords:** Batteries, Batteries

## Abstract

Extremely fast charging (i.e. 80% of storage capacity within 15 min) is a pressing requirement for current lithium-ion battery technology and also affects the planning of charging infrastructure. Accelerating lithium ion transport through the solid-electrolyte interphase (SEI) is a major obstacle in boosting charging rate; in turn, limited kinetics at the SEI layer negatively affect the cycle life and battery safety as a result of lithium metal plating on the electrode surface. Here, we report a γ-ray-driven SEI layer that allows a battery cell to be charged to 80% capacity in 10.8 min as determined for a graphite full-cell with a capacity of 2.6 mAh cm^−2^. This exceptional charging performance is attributed to the lithium fluoride-rich SEI induced by salt-dominant decomposition via γ-ray irradiation. This study highlights the potential of non-electrochemical approaches to adjust the SEI composition toward fast charging and long-term stability, two parameters that are difficult to improve simultaneously in typical electrochemical processes owing to the trade-off relation.

## Abstract (web summary)

Extremely fast charging such as charging 80% of capacity within 15 min is a pressing requirement for current lithium-ion battery technology. Here the authors achieve this by incorporating an artificial solid-electrolyte interphase rich in inorganic components on the graphite electrode.

## Introduction

The maturing of lithium-ion batteries (LIBs) has changed our lives drastically by enabling the widespread consumer uptake of portable electronics and electric vehicles (EVs)^1,2^. Along with the invention of active electrode materials, decades of research has taught us that the solid–electrolyte interphase (SEI) plays a critical role in advancing the performance of LIBs^3–6^. Being electronically insulating, the SEI layer prevents continuous decomposition of the electrolyte while permitting the migration of lithium (Li) ions. Hence, the stability of the SEI layer influences the Coulombic efficiency (CE) of each cycle and consequently the long-term cyclability. Simultaneously, the composition and physicochemical properties of the SEI layer are the main parameters that determine Li ion migration at the interface^7,8^.

Among the various specifications for evaluating a LIB cell, fast charging capability enjoys the highest priority among general EV users as the relatively lengthy charging time is the most significant inconvenience compared to conventional internal combustion vehicles. Fast charging would also have a direct impact on the master plan for the construction of charging infrastructure in society. Nevertheless, improving the charging rate without sacrificing other key electrochemical properties is technically nontrivial. In the current cell configuration of LIBs, the interfacial resistance at the anode has been identified as a major barrier in the way of boosting the charging rate^7,9^. In particular, the consequence of the sluggish ionic diffusion associated with interfacial polarisation is that Li metal is plated on the surface of the anode, compromising the battery safety by creating internal short circuits as well as the cycle life by promoting parasitic side reactions^10^. Thus, developing an SEI layer with high Li-ion diffusivity and enhanced stability has become a target of the utmost priority.

SEI engineering has mainly been driven by introducing additives to the electrolyte that can be decomposed prior to the electrolyte solvents to dictate the primary composition of the SEI layer^11,12^. In this electrochemical process of charging, both the salt and solvents are indiscriminately decomposed by electrochemical reduction, endowing the SEI composition with limited tunability, particularly with respect to the ratio of inorganic to organic components. To overcome this shortcoming, we focused on a different energy source, namely, γ-rays, which are ionising electromagnetic rays of sufficient intensity to break chemical bonds^13^. The approach of employing γ-rays is linked to the rationale that a photochemical process can induce distinct decomposition behaviour between salts and organic solvents, offering the ability to adjust the composition of the resulting SEI. In fact, γ-rays can afford^14^ to excite the electrons of all elements to the vacuum level and drive a wide range of reactions. Notably, γ-rays have been used in industry^15–18^ for the purpose of disinfection, processing of plastics, and crystal colouring, and may thus be adoptable on a practical scale once their effect is confirmed.

Having noted that γ-rays can induce an artificial SEI layer with a favourable composition and also that the composition of the SEI layer has a substantial effect on the charging rate, in this study, we used γ-ray-driven artificial SEI layers for graphite anodes to investigate their electrochemical and physicochemical properties. The graphite anode in conjunction with the photochemically driven SEI layer exhibited superior charging performance, requiring 10.8 min to reach 80% state-of-charge (SOC) for a graphite full-cell with a capacity of 2.6 mAh cm^−2^ while maintaining long-term cyclability without extensive Li metal plating. These findings point to new opportunities for realising highly demanded fast charging by employing non-electrochemical approaches to tune the SEI composition as desired.

## Results

### Electrochemical vs. photochemical SEI

**Fig. 1:**
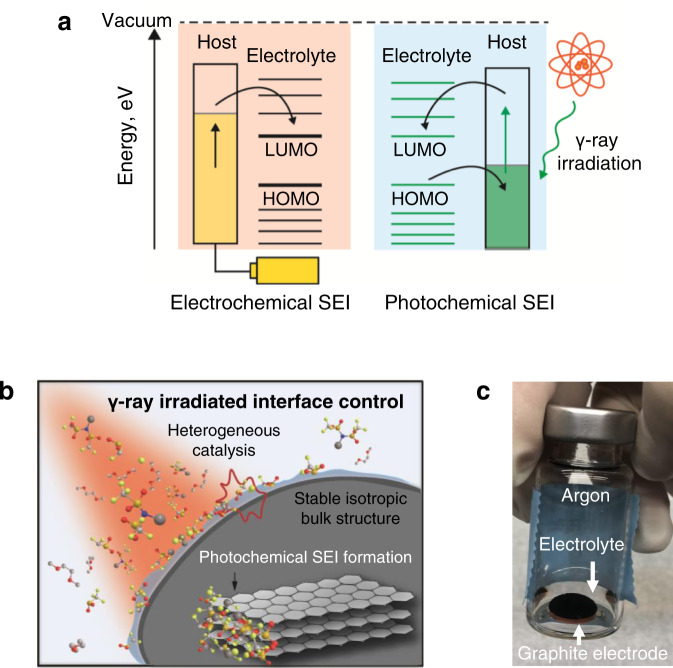
Photochemical SEI formation induced by γ-rays. **a** Distinct energy schemes in the formation of the SEI using electrochemical and photochemical approaches. **b** Interface modification of the graphite electrode induced by γ-ray irradiation. **c** Photograph of the graphite electrode immersed in the electrolyte inside a vial filled with argon for γ-ray irradiation.

Electron transfer in both electrochemical and photochemical processes is illustrated in Fig. 1a. In the electrochemical process, electrons are transferred unidirectionally from graphite to the lowest unoccupied molecular orbital (LUMO) level of the electrolyte during its reduction to form the SEI layer. Thus, both the salts and solvents in the electrolyte are indiscriminately decomposed via the same mechanism, providing compositions with relatively narrow ranges of the components of the SEI layer.

On the other hand, γ-ray irradiation can drive photochemical reactions that involve radical intermediates. Thus, irradiation with γ-rays can preferentially decompose compounds, which are easily converted into radicals^19^, representing improved tunability of the SEI composition; when a salt is designed to favourably form a radical intermediate upon exposure to electromagnetic radiation, a salt-driven SEI composition rich in inorganic components may be induced (Fig. 1b). This reasoning is particularly valid for fluorine (F)-containing salts because halogen elements are generally known to form stable radicals^19,20^. The presence of graphite is also advantageous for inducing a radical from a F-containing salt; being structurally stable based on the isotropic *sp*^2^ bond configuration, the aromatisation network of the graphite surface generates radicals by absorbing photon energy^21^. The generated radicals can be transferred to the electrolyte or vice versa^22^, further enriching the formation of radicals from salts via heterogeneous photocatalytic paths. This photochemical approach enables lithium fluoride (LiF), which was recently identified^23^ to be beneficial for stabilising the interface, to be more aggressively derived from a F-containing salt, thereby enhancing the fast charging capability. In fact, this is the reason why a LiF-rich SEI layer has been pursued by following various strategies^24^.

The compositions of the electrochemically and photochemically driven SEI layers (denoted echem-SEI and photo-SEI, respectively) were investigated using X-ray photoelectron spectroscopy (XPS) analysis (Supplementary Fig. 1). The echem-SEI was formed in a half-cell setting, whereas the photo-SEI was produced by irradiating the graphite electrode immersed in the electrolyte with γ-rays (details appear in the ‘Methods’ section). The XPS results indicate that the compositions of the two SEI layers are conspicuously different although the same electrolyte comprising 1 M lithium hexafluorophosphate (LiPF_6_) in ethylene carbonate/diethyl carbonate (EC/DEC = 50/50 (v/v) with 10 wt% fluoroethylene carbonate (FEC)) was used. The relative ratio of the peak intensities of the F 1*s* and O 1*s* branches, representative of the inorganic-to-organic ratio, reveals that the conventional echem-SEI consists of an organic-inorganic mixture (Supplementary Fig. 1a). For example, the organic components indicated by the C–O–C and RCO_3_Li peaks at 533.6 and 531.7 eV constitute 13.8% and 32.3% of the entire SEI components, respectively. In stark contrast, the photo-SEI is far richer in inorganic components: the LiPF_6_ peak at 688.0 eV (22.4%), Li_*x*_PF_*y*_ peak at 686.9 eV (42.4%), and LiF peak at 685.6 eV (21.7%) (Supplementary Fig. 2). These results are a direct indication that γ-rays can induce an inorganic-rich SEI beyond the range achievable with conventional electrochemical processes.

### γ-Ray-induced photochemical SEI

The photochemical process grants us the freedom to test a variety of electrolyte conditions, obviating the need to adhere to the stringent criteria that apply to typical electrolyte screening, such as a stable potential window, viscosity, polarity, and volatility. The formation of the actual photo-SEI was accomplished by immersing the graphite electrode in various electrolytes and sealing them under argon atmosphere (Fig. 1c), followed by exposure to γ-rays. After testing different salts and irradiation times and focusing on the rate performance (Supplementary Fig. 3), 1 M lithium bis(trifluoromethanesulfonyl)imide (LiTFSI) in EC/DEC (50/50 = v/v) was chosen as the electrolyte for artificial SEI formation. Other F-based lithium salts including LiPF_6_ and LiFSI were also found to produce a LiF-containing SEI layer but with lower LiF content compared with that of LiTFSI (Supplementary Fig. 4). Apart from this, the electrolyte based on LiPF_6_ underwent degradation during long-term storage, which could presumably be ascribed to the uncontrolled reactivity of the generated radicals (Supplementary Fig. 5). The detailed reaction mechanism of each electrolyte is proposed based on the intermediate compounds detected by liquid chromatography–mass spectrometry (LC–MS) analysis (Supplementary Figs. 6 and 7). We thus proceeded to carry out subsequent electrochemical evaluations mainly with this graphite electrode that was treated with 1 M LiTFSI in EC/DEC (50/50 = v/v) and denoted this sample as photo-graphite. We additionally checked whether an artificial SEI layer could be formed by immersing the electrode in the electrolyte (1 M LiTFSI in EC/DEC (50/50 = v/v)) for one day without γ-ray irradiation. As expected, this control sample neither showed any sign of an artificial SEI layer nor did the rate performance improve (Supplementary Fig. 8).

We first performed X-ray diffraction (XRD) analysis to determine whether the γ-ray beam disrupted the crystallinity of graphite. The XRD patterns (Supplementary Fig. 9) verified that the bulk structure of the graphite remained intact, which is attributed to the ability of the isotropic *sp*^2^ bond configuration to restore the bond network even after the formation of radicals. Even once the artificial photo-SEI layer had formed, decomposition of the EC and DEC in the bulk electrolyte could barely be detected by ^1^H- and ^13^C-nuclear magnetic resonance (NMR) spectroscopy (Supplementary Fig. 10). This observation related to the bulk electrolyte, even at a radiation dose of 50 kGy, indicates that the decomposition reaction upon photolysis is concentrated at the interfaces, presumably assisted by the photocatalytic role of graphite.

**Fig. 2:**
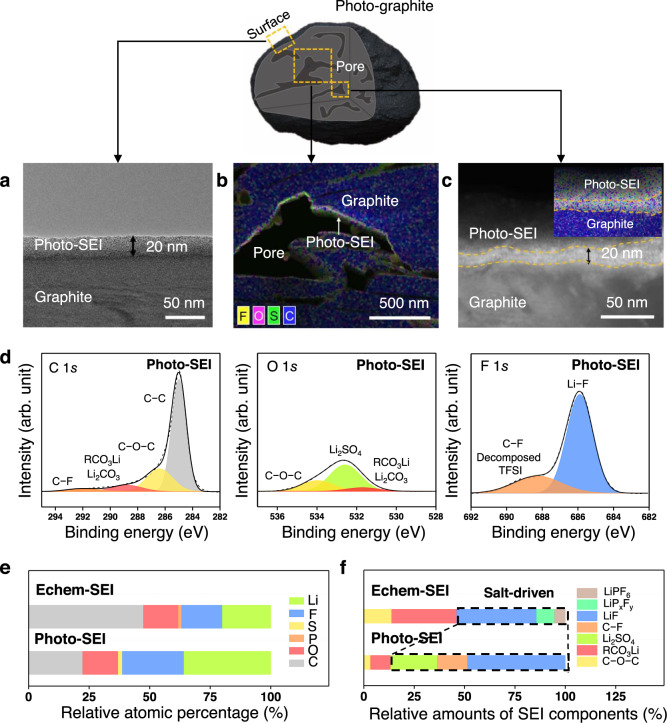
Characterisation of the photo-SEI. **a** Cs-TEM image of the photo-graphite with the photochemically driven SEI layer. **b** Low-magnification EDS elemental map of the photo-SEI inside the pores of the photo-graphite electrode. **c** High-magnification Cs-TEM image of the photo-SEI image and the corresponding EDS map (inset). **d** XPS profiles of the photo-SEI in the C 1*s* (left), O 1*s* (middle) and F 1*s* (right) bands. **e** Relative atomic contents of echem-SEI and photo-SEI. **f** Relative amounts of SEI components in echem-SEI and photo-SEI. The photo-SEI in this figure originates from 1 M LiTFSI in EC/DEC (50/50 = v/v).

The decomposed LiTFSI in EC/DEC on the graphite surface was characterised using various analytical tools. The Cs-corrected transmission electron microscopy (Cs-TEM) image of photo-graphite indicates that the photo-SEI layer was uniformly deposited on the graphite surface with a thickness of ~20 nm (Fig. 2a). The uniform nature of the coating of the photo-SEI layer was further corroborated by energy dispersive spectroscopy (EDS) mapping (Supplementary Fig. 11), which revealed the homogeneous distribution of fluorine originating from the aforementioned F-containing photo-SEI components. The photo-SEI layer was also observed to exist in the inner pores of the graphite particles (Fig. 2b, c), implying that γ-ray irradiation was effective even in the interior of the graphite. This feature would be expected to be beneficial for fast charging that depends on Li ion transport in both intra- and inter-particle space. In addition, although the XPS profile confirmed that LiF had in fact formed (Supplementary Fig. 2b), XRD and selected area electron diffraction (SAED) analyses revealed the LiF in the photo-SEI layer to be mostly amorphous (Supplementary Figs. 12 and  13).

For a more exact analysis, we used highly oriented pyrolytic graphite (HOPG) with a flat surface, which was subjected to the same γ-ray irradiation to yield a photo-SEI layer on the surface. The use of HOPG excludes the curvature effect of graphite powder as well as the influence of binder from the analysis of the SEI layer. According to the XPS analysis (Fig. 2d), the photo-HOPG contained 35.8 at% of lithium, 22.3 at% of carbon, 14.6 at% of oxygen, 1.7 at% of sulfur, and 25.5 at% of fluorine when the portion of C–C bonds related to the graphite is excluded. Importantly, the elemental composition of photo-SEI differed drastically from that of echem-SEI (Fig. 2e) in that photo-SEI had a much higher fluorine content (25.5 vs. 16.7 at%). The F 1*s* profile portrays the distinct chemical nature of the F-containing compounds in the photo-SEI compared with those in the echem-SEI. The photo-SEI has organic- and inorganic-based components in an atomic ratio of 13.3% and 86.7%, respectively, in contrast to 46.1% and 53.8% in the echem-SEI (Fig. 2f). The fluorine and LiF content of the photo-SEI was observed to be consistently high (25.5 at% and relative content of 76.2% in the F 1*s* branch, respectively), driven by one of the most commonly used electrolytes in current LIBs, 1 M LiPF_6_ in EC/DEC (50/50 = v/v) with 10 wt% FEC (Supplementary Figs. 1 and 2). We anticipate the high LiF content to reflect the preferential decomposition of the TFSI anion by engaging its radical intermediate products. Apparently, the relative chemical stability of electrolyte solvents upon photolysis plays a role in achieving the inorganic-rich photo-SEI layer without an undesirable amount of by-products.

**Fig. 3:**
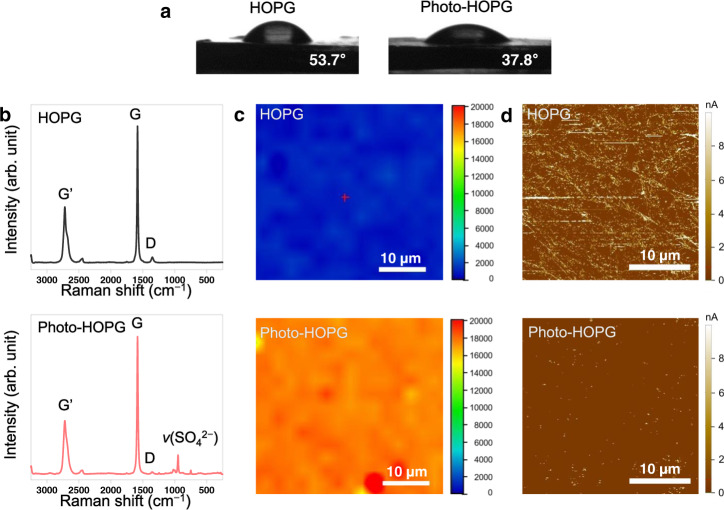
Characterisation of bare and photo-HOPG. **a** Contact angle measurements of bare HOPG and photo-HOPG. **b** Raman spectra of bare HOPG and photo-HOPG. **c** Raman 2D mapping images of bare HOPG and photo-HOPG at 950 cm^–1^. **d** AFM current mapping images of HOPG and photo-HOPG with 0.5 V bias.

The formation of the inorganic-rich layer on the HOPG surface has a marked impact on its surface characteristics. When deionised water was dropped on the surface, the contact angles of bare and photo-HOPG were 53.7° and 37.8°, respectively (Fig. 3a). The more hydrophilic surface of photo-HOPG is ascribed to the ionic bond in LiF. The inorganic-rich interface was also characterised by Raman spectroscopy (Fig. 3b, c). Compared to the Raman profile of bare HOPG, which exhibited the well-known graphitic signature composed of G’, G and D bands^25^ (Fig. 3b, top), the Raman profile of photo-HOPG displayed a new peak at 950 cm^−1^ corresponding to the *A*_1_ vibration mode of the SO_4_^2−^ anion (Fig. 3b, bottom), another indication of the inorganic-rich interface driven by lithium salt. Utilising this signature at 950 cm^−1^, the homogeneous distribution of the SO_4_^2−^ anion in the inorganic-rich SEI layer was verified in an area of 30 × 30 μm^2^ using 2D Raman mapping (Fig. 3c). The surface coverage of the insulating LiF-rich SEI was further revealed by atomic force microscopy (AFM) current mapping such that the current measured for photo-HOPG over the entire area of 30 × 30 μm^2^ was clearly lower compared with that of bare HOPG (Fig. 3d and Supplementary Fig. 14). Here, we need to stress that it is generally very difficult to uniformly spread inorganic components over an SEI layer owing to the high rigidity of their crystals^26^. In this sense, the uniform coverage of the LiF-rich photo-SEI is remarkable, representing a unique opportunity associated with the photochemically driven process.

### Electrochemical tests on the photo-graphite cell

**Fig. 4:**
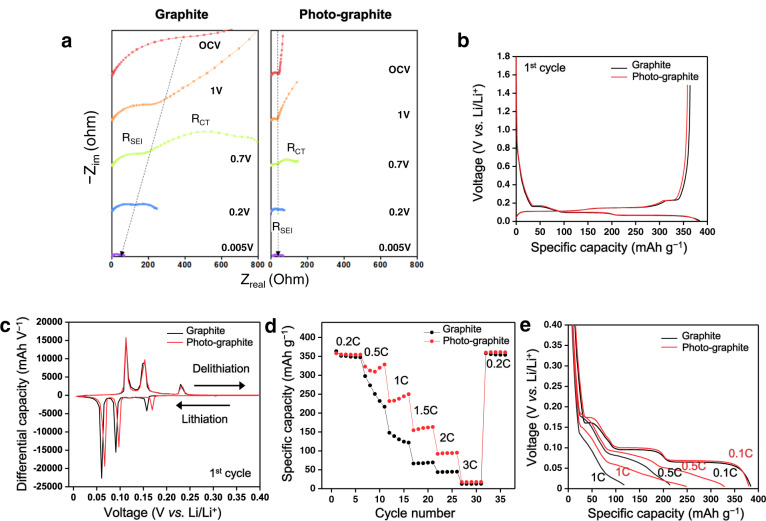
Electrochemical measurement of graphite half-cells. **a** EIS profiles of bare graphite and photo-graphite half-cells during the initial cycles. **b** Initial charge and discharge profiles of bare graphite and photo-graphite half-cells when scanned at 0.1 C (35 mA g^−1^). **c** d*Q*/d*V* profiles of bare graphite and photo-graphite half-cells during the initial cycles at 0.1 C (35 mA g^−1^). **d** Rate capability results of bare graphite and photo-graphite half-cells. **e** Comparative discharge profiles at 0.1 C, 0.5 C and 1 C. The active material loading for these results was 8.3 mg cm^−2^.

Based on the consensus^8^ that the interfacial ionic resistance is a major hurdle in the way of realising fast charging, electrochemical impedance spectroscopy (EIS) was used to investigate the interfacial resistance in a half-cell (Fig. 4a). Notably, the ionic transport resistance at the interface of the cell was 10 times lower after the photochemical treatment, pointing to the fact that the modified SEI layer has a dramatic impact on the ionic transport at the interface. Remarkably, the SEI resistance (R_SEI_) and charge transfer resistance (R_CT_) of the photo-graphite cell remained quite low throughout the potential range in which lithiation occurs (Fig. 4a, right) as an indication of the formation of a highly compact artificial SEI layer. This observation contrasts that of the bare graphite counterpart in which both R_SEI_ and R_CT_ decreased prominently during the same course of lithiation (Fig. 4a, left)^27^.

Next, the electrochemical performance was evaluated under the half-cell setting. In the first cycle, the photo-graphite cell exhibited charge–discharge profiles that were almost identical to those of its bare graphite counterpart (Fig. 4b), indicating that the main Li storage chemistry of graphite was largely maintained. This electrochemical behaviour is contrary to that of previous studies, which demonstrated improved rate performance at the expense of the original storage mechanism; particularly, chemically treated graphite offers enhanced rate capability, but the well-defined flat plateaus at low potentials are greatly impaired^28^. The differential capacity profiles of the two cells were also quite consistent with respect to the positions of the main peaks (Fig. 4c). Nonetheless, the degree of polarisation of these peaks decreased conspicuously even at a low scan rate of 0.1 C (35 mA g^−1^).

To examine the direct effect of the photo-SEI on the rate performance, the specific capacity was monitored by increasing the C-rate (Fig. 4d). When operated in constant current (CC) mode for both lithiation and delithiation, although both the bare and photo-graphite cells had similar specific lithiation capacities of nearly 360 mAh g^−1^ at 0.2 C, the photo-graphite cells exhibited markedly greater capacities at higher C-rates. Whereas the photo-graphite cells retained 328.5, 249.9, 163.2 and 95.2 mAh g^−1^ at 0.5 C, 1 C, 1.5 C and 2 C, respectively, the bare graphite cell preserved only 216.8, 121.9, 69.5 and 44.9 mAh g^−1^ at the same C-rates. The lithiation profiles of the two cells elucidate the distinct rate performances (Fig. 4e). Consistent with the dQ/dV results in Fig. 4c, the overpotentials of the photo-graphite cell were evidently lower, even at the low scan rate of 0.1 C, compared to those of the bare graphite cell, although both cells displayed a clear staging effect^29^. The charging (lithiation) profiles became increasingly distinct as the C-rate increased. In CC mode, the bare graphite cell reached the low cut-off voltage of 0.005 V vs. Li/Li^+^ much earlier at a given C-rate, revealing its limited charging (lithiation) capability. In fact, as a consequence of the larger overpotential of the bare graphite cell, its charging profile at 0.5 C is located near the low cut-off voltage, explaining the rapid decrease in capacity with cycling at the given C-rate (Fig. 4d), i.e. the capacity is highly sensitive to even a small increase in the resistance. These results unveil the effect of the photochemically driven SEI layer on the charging rate in relation to the interfacial resistance. This charging capability is superior to most of those reported in the literature (Supplementary Table 1) even though different electrode conditions make it difficult to compare the performance directly.

**Fig. 5:**
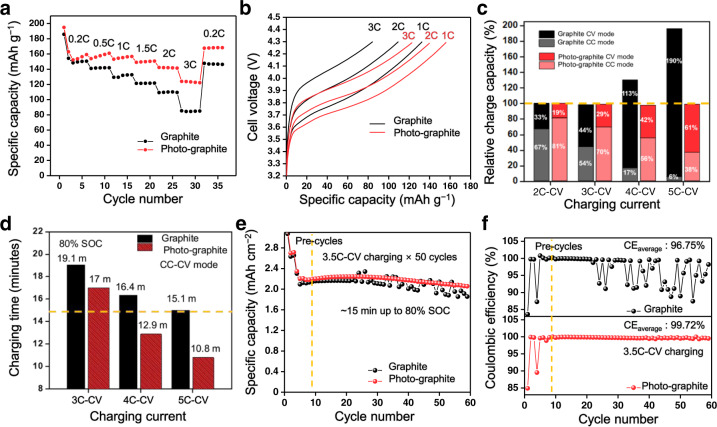
Electrochemical measurements of photo-graphite full-cell. **a** Rate capability test results of bare graphite and photo-graphite full-cells. **b** Charging profile comparison at 1 C, 2 C and 3 C. **c** Relative charge capacity for CC and CV modes at various charging rates. **d** Charging time to reach the 80% SOC at various charging rates. **e** Cycling performance of both types of full-cells at 3.5 C in CC-CV mode. **f** Comparison of the Coulombic efficiency at 3.5 C in CC-CV mode. Both types of full-cells have an areal capacity of 2.6 mAh cm^–2^ and N/P ratio of 1.1.

The superior charging capability of the photo-graphite was subsequently assessed in full-cells in which it was paired with a lithium nickel manganese cobalt oxide (LiNi_0.6_Co_0.2_Mn_0.2_O_2_ or NCM622) cathode (active material:Super P:binder = 95:3:2 by weight). Figure 5a, b presents the charging capacities at different C-rates in CC mode and their corresponding charging profiles, respectively. Consistent with the half-cell results, the charging rate performance of the photo-graphite full-cell improved significantly over the range of C-rates by lowering the overpotential before the top cut-off potential, 4.3 V, was reached. The distinct full-cell performance revalidates the remarkable contribution of the photo-SEI toward boosting the charging capability.

The charging condition was also extended to include the constant voltage (CV) mode to benchmark the practical CC-CV operation. Before the fast charging capability was evaluated, the cells were cycled at 1C-CV to examine their basic electrochemical performance. Both types of cells were observed to deliver robust cycling performance for 300 cycles (Supplementary Fig. 15), thus validating that they were fabricated appropriately. The fast-charging tests were conducted at room temperature at various charging rates from 2 C to 5 C while the discharging rate was held constant at 0.2 C to focus on the charging process. As illustrated in the bar graph in Fig. 5c, the photo-graphite full-cell delivered a much higher percentage of the charging capacity in CC mode compared with its bare graphite full-cell counterpart. Interestingly, the bare graphite full-cell exceeded 100% of full capacity (defined on the basis of the discharging capacity at the 0.2 C-rate) at the 4 C-rate or above such that its relative charging capacities at 4C-CV (10.5 mA cm^−2^) and 5C-CV (13.2 mA cm^−2^) were 130% and 196%, respectively. In contrast, similar overcharging behaviour was never obvious for the photo-graphite full-cell (Supplementary Fig. 16). The observed overcharging behaviour is attributed to Li metal plating on the graphite electrode owing to severe interfacial polarisation^30–33^. The prevention of Li metal plating on the photo-graphite anode was verified by monitoring the anode voltage in a three-electrode Swagelok cell (Supplementary Fig. 17).

The charging time required to reach 80% SOC in CC-CV mode is presented in Fig. 5d. An increase in the C-rate, apparently, consistently shortened the time required to reach this state for both cells. However, the extent to which the charging time decreased was more substantial with the photo-graphite full-cell. For example, at the charging rate of 4 C, the time required for 80% charging was 16.4 and 12.9 min for the bare and photo-graphite full-cells, respectively. These charging times were shortened to 15.1 and 10.8 min, respectively, at the charging rate of 5 C. For the reference, reaching 80% charging within 15 min is referred^8^ to as extremely fast charging (XFC), a formidable target that has been set by the battery community. In this sense, the fast charging performance delivered by the photo-SEI layer is remarkable.

We then assessed the sustainability of cycling under XFC conditions because it is widely accepted^30^ that Li metal plating, which tends to be accelerated during fast charging, could deteriorate the cycle life severely. To this end, the long-term cyclability was tested at 3.5 C in CC-CV mode, which corresponds to a charge time of approximately 15 min. The cycling stability of the two full-cells (Fig. 5e) turned out to be drastically different. Whereas the bare graphite full-cell underwent capacity fluctuation accompanied by overcharging at certain cycling points, the cycling behaviour of the photo-graphite full-cell was quite stable throughout the 50 cycles and this was accompanied by lower interfacial resistance (Supplementary Fig. 18). As discussed above, the overcharging of the bare graphite full-cell is attributed to uncontrolled Li metal plating originating from its large polarisation. The distinct cycling stability was also reflected in the CE (Fig. 5f). In the case of the bare graphite full-cell, destabilisation of the CE commenced at around the 24th cycle, delivering an average value of 96.75% for 50 cycles. In contrast, the CE of the photo-graphite full-cell was far more stable with an average value of 99.72% under the same cycling conditions. The superior cyclability of the photo-graphite full-cell was maintained for 200 cycles (Supplementary Fig. 19). Field emission scanning electron microscope (FE-SEM) observation after 100 cycles of 3.5 C charging in CC-CV mode revealed that the surface of the bare graphite electrode was completely covered with plated metallic Li, whereas the surface of the photo-graphite was much cleaner (Supplementary Figs. 20 and 21). In the same vein as the fast charging capability, the low interfacial resistance of the photochemically driven SEI also permits low-temperature operation, another formidable challenge faced by current LIBs. When charging at 1C-CV at 0 °C, where Li ion diffusion becomes considerably sluggish, the photo-graphite delivered superior capacity retention and CE compared to those of the bare graphite (Supplementary Fig. 22). The photo-graphite full-cell exhibited higher cycling performance even at the high temperature of 60 °C in 6C-CV charging mode (Supplementary Fig. 23). Importantly, the graphite loading, 8.3 mg cm^−2^ or 2.9 mAh cm^−2^, in the present study is within the range adopted for cells used in practice.

**Fig. 6:**
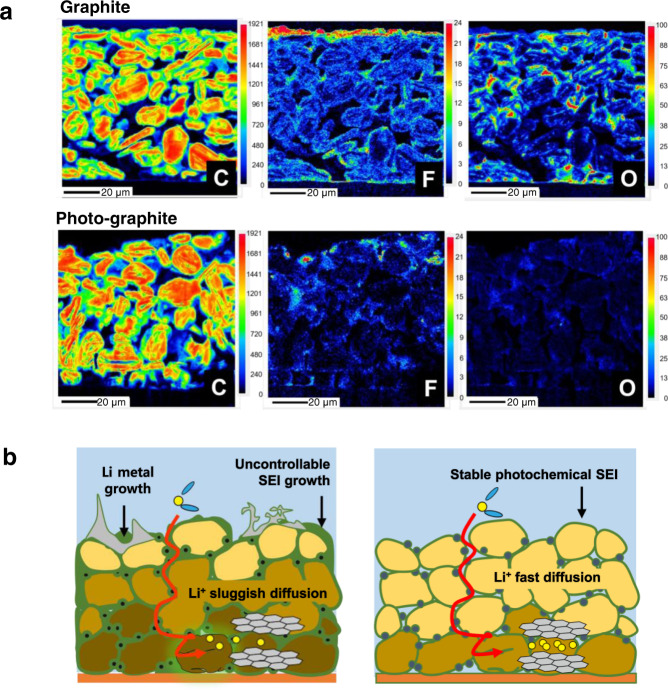
Ex situ characterisation of both types of graphite electrodes. **a** FE-EPMA mapping images of cross-sections of bare graphite and photo-graphite after rate capability tests. **b** Schematic illustrations of problems expected with fast charging and the advantageous effect of the photochemical SEI.

The most daunting challenge that would need to be overcome to warrant fast charging is the prohibition of Li metal plating on the graphite electrode, an outcome of the sluggish kinetics of Li ion intercalation^31–33^. Li metal plating has a catastrophic effect on both the cycle life and safety as it causes unwanted parasitic reactions with the electrolyte and results in internal short circuits^10,30^. With this rationale in mind, post-mortem field emission-electron probe microanalysis (FE-EPMA) was conducted on the cross-sections of both types of graphite electrodes before and after the rate tests. Before the rate tests, negligible signals were detected with respect to oxygen and fluorine (Supplementary Fig. 24). After the rate tests, however, the SEI of the bare graphite electrode was detected to have grown massively in size according to the signals of oxygen and fluorine (Fig. 6a, top). In particular, a thick F-containing SEI layer was observed to cover the electrode as a result of electrolyte decomposition on the plated metallic Li. Apart from the deposits on the surface, oxygen-containing SEI layers were found in the inner voids of the electrode as additional evidence of relatively uncontrolled SEI formation. Contrary to this, the fluorine and oxygen signals associated with the photo-graphite electrode were much weaker for both the exterior and interior of the electrode owing to suppressed Li metal plating (Fig. 6a, bottom).

The series of electrochemical and physicochemical properties of both types of graphite electrodes reveal the importance of SEI components, particularly in terms of the relative inorganic-to-organic content. In a reductive environment, the organic components in the SEI layer are vulnerable to being transformed into radical intermediates, which tend to undergo subsequent chemical reactions with each other^34,35^. The radical intermediates can also serve as bridges to promote electron transfer beyond the feasible range in typical radical-free intermolecular space, heightening the chance of the SEI layer experiencing growth. The thickening of the SEI layer during cycling impedes the intercalation of Li ions into the graphite (Fig. 6b, left), accelerating Li metal plating on the electrode surface at high C-rates and worsening the cycling performance. Overall, an SEI layer with an organic-rich composition is unfavourable for fast charging with robust cycling. In contrast, an abundance of chemically stable inorganic components in the SEI layer is mostly insulating and effectively prevents electron transfer toward parasitic reactions, allowing the SEI layer to remain thin during cycling^34,36^ and supporting fast charging operation without severe Li metal plating (Fig. 6b, right). The absence of overcharging in the photo-graphite full-cell can be understood in the same context.

## Discussion

Although the limited interfacial kinetics of Li ions have long been noted to be the main obstacle in the way of XFC, it has not been easy at all to find a solution that does not sacrifice the cycle life and safety. This limitation is attributed to the lack of controllability of the decomposition behaviour of the salts and solvents in the electrolyte, particularly their relative contributions in an electrochemically reductive environment. The γ-ray irradiation introduced in this study accelerates the preferential decomposition of the F-containing salt to induce the formation of a LiF-rich SEI layer. This inorganic-rich SEI layer promotes Li ion diffusion at the interface and remains stable as a result of the insulating nature of the inorganic compounds, thus enabling a long-term cycle life while achieving XFC in 10.8 min for 80% charging. The proposed approach involving the use of γ-rays may not be immediately adoptable in the current manufacturing line of LIBs. However, it offers useful insights that may stimulate possible research directions for electrolyte engineering toward XFC. These insights may also be applicable to other LIB electrodes that experience interfacial instability.

## Methods

### Materials

Mesocarbon microbeads (MCMB) graphite and NCM622 were used as active materials for the anodes and cathodes, respectively. HOPG was purchased from Alfa Aesar. LiTFSI and lithium bis(fluorosulfonyl)imide (LiFSI) were purchased from Sigma-Aldrich, USA. *N*-methyl-2-pyrrolidone (NMP) was purchased from JUNSEI, Japan. Super P was purchased from Timcal, Switzerland. The electrolyte containing 1 M LiPF_6_ in EC/DEC (50/50 = v/v) with 10 wt% FEC was purchased from Wellcos Corporation, South Korea. Styrene-butadiene rubber (SBR, Zeon) and sodium carboxy methyl cellulose (CMC, Sigma-Aldrich) binders were used for the graphite electrodes. Poly(vinylidene fluoride) (PVDF, Kynar) binder was used for the NCM622 electrodes.

### Physicochemical characterisation

XPS results were recorded on an AXIS-His (KRATOS, U.K.) spectrometer after etching with an argon ion beam (2 kV) for 20 s. The relative contents of the SEI components were quantified by integrating the corresponding peaks of the O 1*s* and F 1*s* branches. FE-SEM (JSM-7600F, JEOL, Japan) was used to capture images of the morphology of electrodes. XRD analysis was performed in the 2θ range of 5–80° using a Smart Lab instrument (Rigaku, Japan). Cs-TEM (JEM-ARM200F, JEOL, Japan) was employed to observe cross-sections of the photo-SEI after cross-sectional milling using a focused ion beam (Helios G4, Thermo Fisher, USA). Contact angle measurements were performed using DSA100 (Kruss, Germany) by dropping 5 µL of deionised water on the sample surface. Raman spectra were recorded in the wavenumber range of 250–3250 cm^−1^ using a RAMAN spectrometer II (DXR2xi, Thermo Fisher, USA). AFM analysis was performed using NX-10 instrumentation (Park Systems, South Korea) at the Research Institute of the Advanced Materials Research Center (RIAM) at Seoul National University to obtain the electronic conductivity map by applying bias of 0.5 V. The reaction intermediates during γ-ray irradiation were identified by LC–MS using a Ultimate 3000 (Thermo Scientific, USA) instrument equipped with a Waters Cortex T3 column and a Triple TOF 5600 (AB Sciex, USA) instrument at the National Instrumentation Center for Environmental Management (NICEM) at Seoul National University. ^1^H NMR and ^13^C NMR analyses were conducted using a 500 MHz NMR AvanceIII-500 instrument (Bruker, Germany) at the National Centre for Inter-university Research Facilities (NCIRF) at Seoul National University. FE-EPMA analysis was carried out on a JXA-8530F instrument (JEOL, Japan) to study the cross-sections of the graphite electrodes after ion-beam milling (6 kV, 400 mA, IM4000, Hitachi, Japan).

### Preparation of electrodes

The mass loading of graphite was 8.3 mg cm^−2^, corresponding to areal capacity of 2.9 mAh cm^−2^ when measured at 0.2 C. To fabricate the graphite electrodes, an aqueous slurry consisting of graphite, Super P and SBR-CMC binder in a weight ratio of 93:3:2:2 was cast on copper foil using the doctor blade method, followed by a drying step at 60 °C under vacuum. To fabricate the NCM622 cathodes, a slurry consisting of NCM622, Super P and PVDF binder in a weight ratio of 95:3:2 was cast on aluminium foil, followed by drying at 60 °C under vacuum. The slurry solvent was NMP, and the areal capacity of the NCM622 cathode was 2.6 mAh cm^−2^ when measured at 0.2 C.

The photo-graphite was prepared using the following irradiation process. First, the graphite electrode prepared with the aforementioned procedure was immersed in a crimp-cap vial containing 1 M LiTFSI in EC/DEC (50/50 = v/v), and this vial was sealed under argon (Fig. 1c). This vial was then transferred to a γ-ray irradiator (MDS Nordion, Canada) at the Korea Atomic Energy Research Institute and subjected to γ-ray irradiation at an irradiation rate of 2 kGy h^−1^ with a total radiation dose of 50 kGy from cobalt-60 sources. The processed vials were dismantled inside a glovebox and the electrodes were washed with DEC several times.

### Electrochemical characterisation

All electrochemical measurements were performed using CR2032 coin-type cells that were assembled in an Ar-filled glove box. The diameter of the electrode used in all the cells was 12 mm, and polyethylene (PE) (SK Innovation, South Korea) and 60 µL of 1 M LiPF_6_ in EC/DEC (50/50 = v/v) with 10 wt% FEC were used as the separator and electrolyte, respectively. Prior to actual cycling, a 6 h rest step was programmed to allow the electrolyte sufficient time for soaking. A pre-cycle with a charging process (0.1 C, CC mode) and subsequent discharging process (0.1 C, CC mode) was invoked before the cycling and rate performance tests. For the half-cell rate performance tests, a graphite electrode was assembled with punched Li metal foil and was lithiated at various C-rates (0.2, 0.5, 1.0, 1.5, 2.0 and 3.0 C) with the cut-off voltage of 0.005 V vs. Li/Li^+^. The delithiation rate was held constant at 0.2 C to focus on the effect of the charging (lithiation) performance. Both charging and discharging were processed in CC mode using a battery cycler (WBCS 3000, Wonatech, South Korea). EIS measurements were conducted using a potentiostat (VSP, Bio-Logic, France) over the frequency range from 1 MHz to 1 Hz. To measure the ionic resistance for various SOC states, half-cells were charged (lithiation of graphite) at 0.1 C and paused at 1 V, 0.7 V, 0.2 V and 0.005 V to record the impedance. For the rate capability tests of the full-cells, the N/P ratio, defined by the total anode capacity over the total cathode capacity, was set to 1.1, and the areal capacity of each full-cell was 2.6 mAh cm^−2^. The rate tests were conducted in the potential range of 2.7–4.3 V in CC mode for both charging and discharging. The charging rate was varied from 0.2 C to 3 C while the discharging rate remained constant at 0.2 C. The fast charging capability tests were carried out by cycling the full-cells in CC-CV mode for charging (i.e. 2C-CV, 3C-CV, 4C-CV, 5C-CV) and in CC mode for discharging at 0.2 C. For long-term CC-CV tests, charging was at 3.5C-CV with a cut-off voltage of 4.2 V and discharging was at 0.5 C in CC mode after pre-cycles of 0.1 C (1 cycle to 4.3 V), 1 C, 2 C and 3 C (3 cycles each to 4.2 V). To monitor the potentials of the anode and cathode separately, a three-electrode Swagelok cell consisting of an NCM622 working electrode, a graphite counter electrode, and a Li metal reference electrode was prepared. With this cell, the end of the charging step was defined such that the potential of the working electrode reached 4.25 V (vs. the reference electrode). To evaluate the cyclability at low temperature, full-cells were cycled at 1C-CV for charging and 0.2 C for discharging in the potential range of 2.7–4.2 V at 0 °C after a pre-cycle at 0.1 C in the potential range of 2.7–4.3 V at 25 °C. High-temperature testing was conducted by cycling the full-cells in 6C-CV mode for charging and 1 C for discharging in the potential range of 2.7–4.2 V at 60 °C after the pre-cycle at 0.1 C at 25 °C.

## Supplementary information

Supplementary information.

## Data Availability

The data that support the plots within this paper and other findings of this study are available from the corresponding author upon reasonable request.

## References

[1] Armand M, Tarascon J-M (2008). Building better batteries. Nature.

[2] Goodenough JB, Park K-S (2013). The Li-ion rechargeable battery: a perspective. J. Am. Chem. Soc..

[3] Xu K (2014). Electrolytes and interphases in Li-ion batteries and beyond. Chem. Rev..

[4] Xu K, Wang C (2016). Widening voltage windows. Nat. Energy.

[5] Xu K (2004). Nonaqueous liquid electrolytes for lithium-based rechargeable batteries. Chem. Rev..

[6] Xu K (2017). Manipulating interphases in batteries. Natl Sci. Rev..

[7] Xu K, Cresce AV, Lee U (2010). Differentiating contributions to “ion transfer” barrier from interphasial resistance and Li+ desolvation at electrolyte/graphite interface. Langmuir.

[8] Liu Y, Zhu Y, Cui Y (2019). Challenges and opportunities towards fast-charging battery materials. Nat. Energy.

[9] Persson K (2010). Lithium diffusion in graphite carbon. J. Phys. Chem. Lett..

[10] Aurbach D, Zinigrad E, Cohen Y, Teller H (2002). A short review of failure mechanisms of lithium metal and lithiated graphite anodes in liquid electrolyte solutions. Solid State Ion..

[11] Winter M, Barnett B, Xu K (2018). Before Li ion batteries. Chem. Rev..

[12] Xu K (2009). Whether EC and PC differ in interphasial chemistry on graphitic anode and how. J. Electrochem. Soc..

[13] Nordlund K (2018). Primary radiation damage: a review of current understanding and models. J. Nucl. Mater..

[14] Xu Z (2013). Nano-structure and property transformations of carbon systems under γ-ray irradiation: a review. RSC Adv..

[15] Tomita K, Sugimoto S (1977). A commercial γ-ray irradiation plant in Japan. Radiat. Phys. Chem..

[16] Nassau K, Prescott BE (1975). Blue and brown topaz produced by gamma irradiation. Am. Mineral..

[17] Rouif S (2005). Radiation cross-linked polymers: recent developments and new applications. Nucl. Instrum. Methods Phys. Res., Sect. B.

[18] Hasanain F, Guenther K, Mullett WM, Craven E (2014). Gamma sterilization of pharmaceuticals—a review of the irradiation of excipients, active pharmaceutical ingredients, and final drug product formulations. PDA J. Pharm. Sci. Technol..

[19] Mincher BJ, Wishart JF (2014). The radiation chemistry of ionic liquids: a review. Solvent Extr. Ion-. Exch..

[20] Hioe J, Zipse H (2010). Radical stability and its role in synthesis and catalysis. Org. Biomol. Chem..

[21] Flores-Rojas GG, Lopez-Saucedo F, Bucio E (2020). Gamma-irradiation applied in the synthesis of metallic and organic nanoparticles: a short review. Radiat. Phys. Chem..

[22] Zhu S, Wang D (2017). Photocatalysis: basic principles, diverse forms of implementations and emerging scientific opportunities. Adv. Energy Mater..

[23] Wang C, Meng YS, Xu K (2019). Fluorinating interphases. J. Electrochem. Soc..

[24] Suo L (2018). Fluorine-donating electrolytes enable highly reversible 5-V-class Li metal batteries. Proc. Natl Acad. Sci. USA.

[25] Pimenta MA (2007). Studying disorder in graphite-based systems by Raman spectroscopy. Phys. Chem. Chem. Phys..

[26] Lorandi F (2021). Comparative performance of ex situ artificial solid electrolyte interphases for Li metal batteries with liquid electrolytes. iScience.

[27] Steinhauer M, Risse S, Wagner N, Friedrich KA (2017). Investigation of the solid electrolyte interphase formation at graphite anodes in lithium-ion batteries with electrochemical impedance spectroscopy. Electrochim. Acta.

[28] Cheng Q (2017). Graphene-like-graphite as fast-chargeable and high-capacity anode materials for lithium ion batteries. Sci. Rep..

[29] Ohzuku T, Iwakoshi Y, Sawai K (1993). Formation of lithium-graphite intercalation compounds in nonaqueous electrolytes and their application as a negative electrode for a lithium ion (shuttlecock) cell. J. Electrochem. Soc..

[30] Yang L (2014). Lithium deposition on graphite anode during long-term cycles and the effect on capacity loss. RSC Adv..

[31] Liu Q (2016). Understanding undesirable anode lithium plating issues in lithium-ion batteries. RSC Adv..

[32] Ouyang M (2015). Overcharge-induced capacity fading analysis for large format lithium-ion batteries with Li_*y*_Ni_1/3_Co_1/3_Mn_1/3_O_2_ + Li_*y*_Mn_2_O_4_ composite cathode. J. Power Sources.

[33] Smart MC, Ratnakumar BV (2011). Effects of electrolyte composition on lithium plating in lithium-ion cells. J. Electrochem. Soc..

[34] An SJ (2016). The state of understanding of the lithium-ion-battery graphite solid electrolyte interphase (SEI) and its relationship to formation cycling. Carbon.

[35] Peled E, Menkin S (2017). Review—SEI: past, present and future. Electrochem. Soc..

[36] He M, Guo R, Hobold GM, Gao H, Gallant BM (2020). The intrinsic behavior of lithium fluoride in solid electrolyte interphases on lithium. Proc. Natl Acad. Sci. USA.

