# Supplementary material for: Photochemically driven solid electrolyte interphase for extremely fast-charging lithium-ion batteries
Source: Nat Commun. 2021 Nov 23;12:6807. doi: 10.1038/s41467-021-27095-w (PMC8611023; doi:10.1038/s41467-021-27095-w)
Supplement: Supplementary file 1 — Supplementary information. [file 41467_2021_27095_MOESM1_ESM.pdf]

Photochemically driven solid electrolyte interphase for extremely fast-charging lithium-ion batteries

Minsung Baek[1], Jinyoung Kim[1], Jaegyu Jin[2] and Jang Wook Choi[1,3]*

[1]School of Chemical and Biological Engineering and Institute of Chemical Processes, Seoul National University, 1 Gwanak-ro, Gwanak-gu, Seoul 08826, Republic of Korea

[2]Institute of Battery Technology, SK Innovation, 325 Exporo, Yuseong-gu, Daejeon 34124, Republic of Korea.

[3]Department of Materials Science and Engineering, Seoul National University, 1 Gwanak-ro, Gwanak-gu, Seoul 08826, Republic of Korea

*E-mail: jangwookchoi@snu.ac.kr

Supplementary Figures

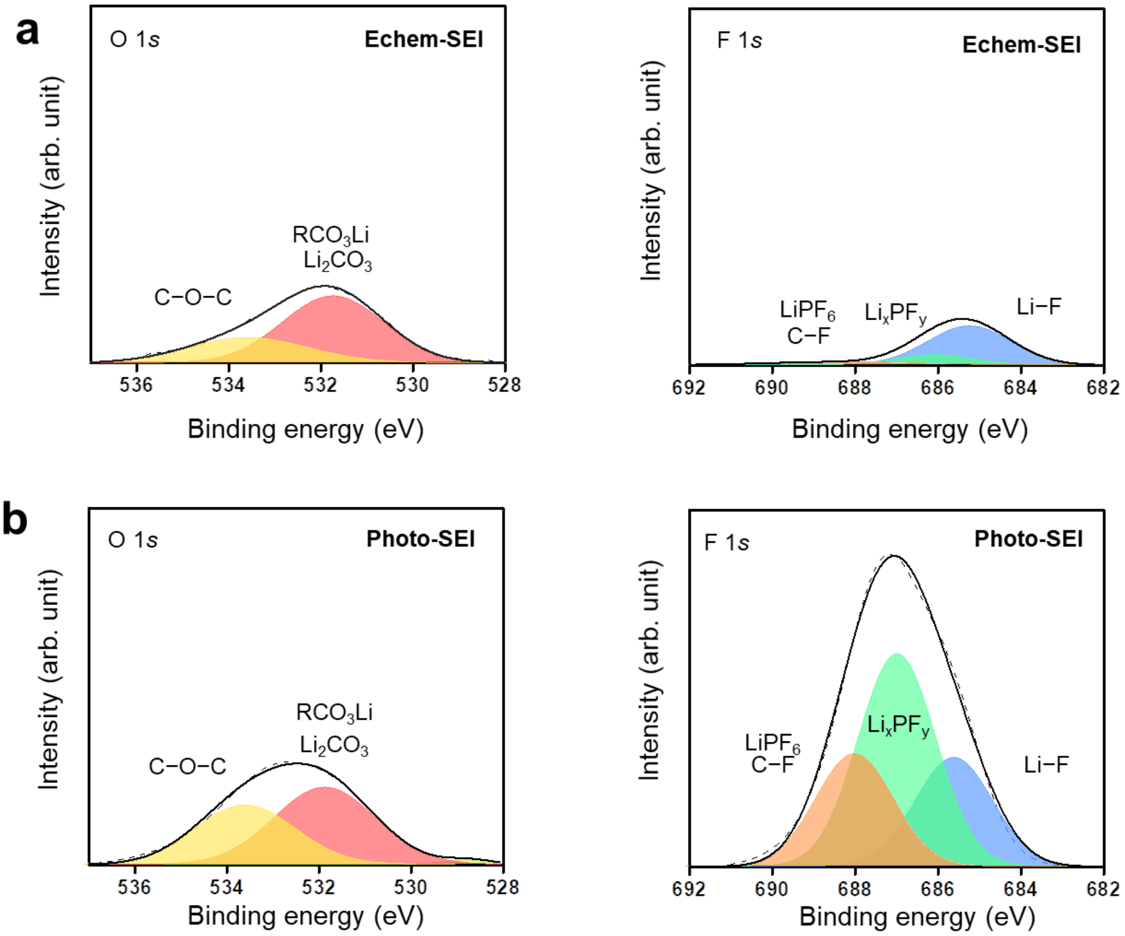

Supplementary Figure 1 | XPS profiles of (**a**) echem-SEI and (**b**) photo-SEI in O 1*s* and F 1*s* branches. 1 M $LiPF_6$ in EC/DEC (50/50=v/v) with 10 wt% FEC was commonly used as electrolyte for both samples.

a

b

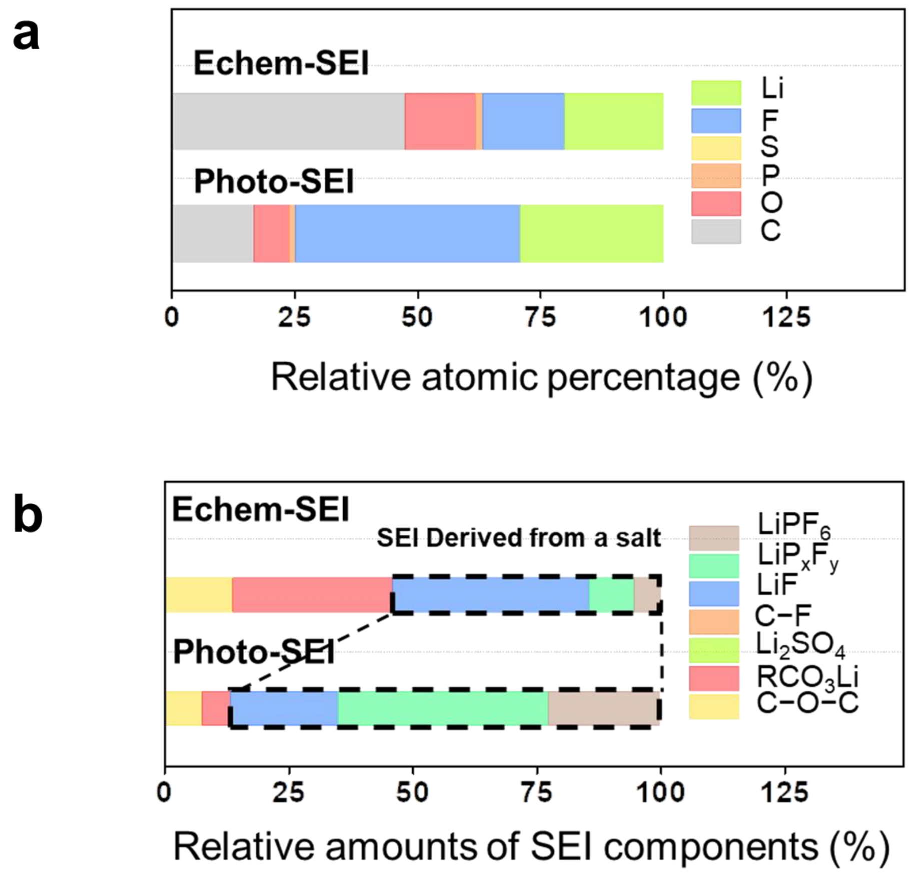

Supplementary Figure 2 | a, Relative atomic contents of echem-SEI and photo-SEI. **b**, Relative amounts of SEI components in echem-SEI and photo-SEI. 1 M $LiPF_6$ in EC/DEC (50/50=v/v) with 10 wt% FEC was commonly used for both samples.

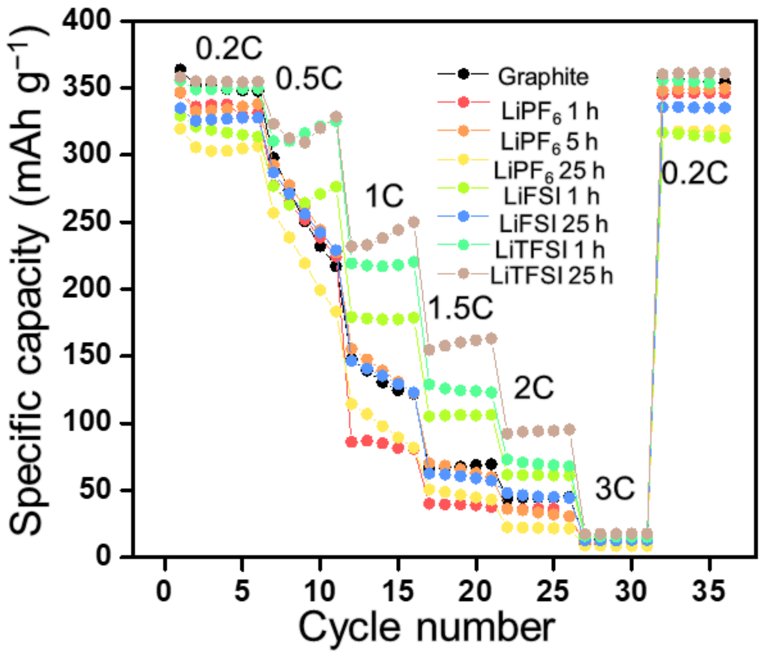

Supplementary Figure 3 | Rate capability results of the photo-graphite half-cells using various irradiation conditions with regard to the lithium salt and irradiation time.

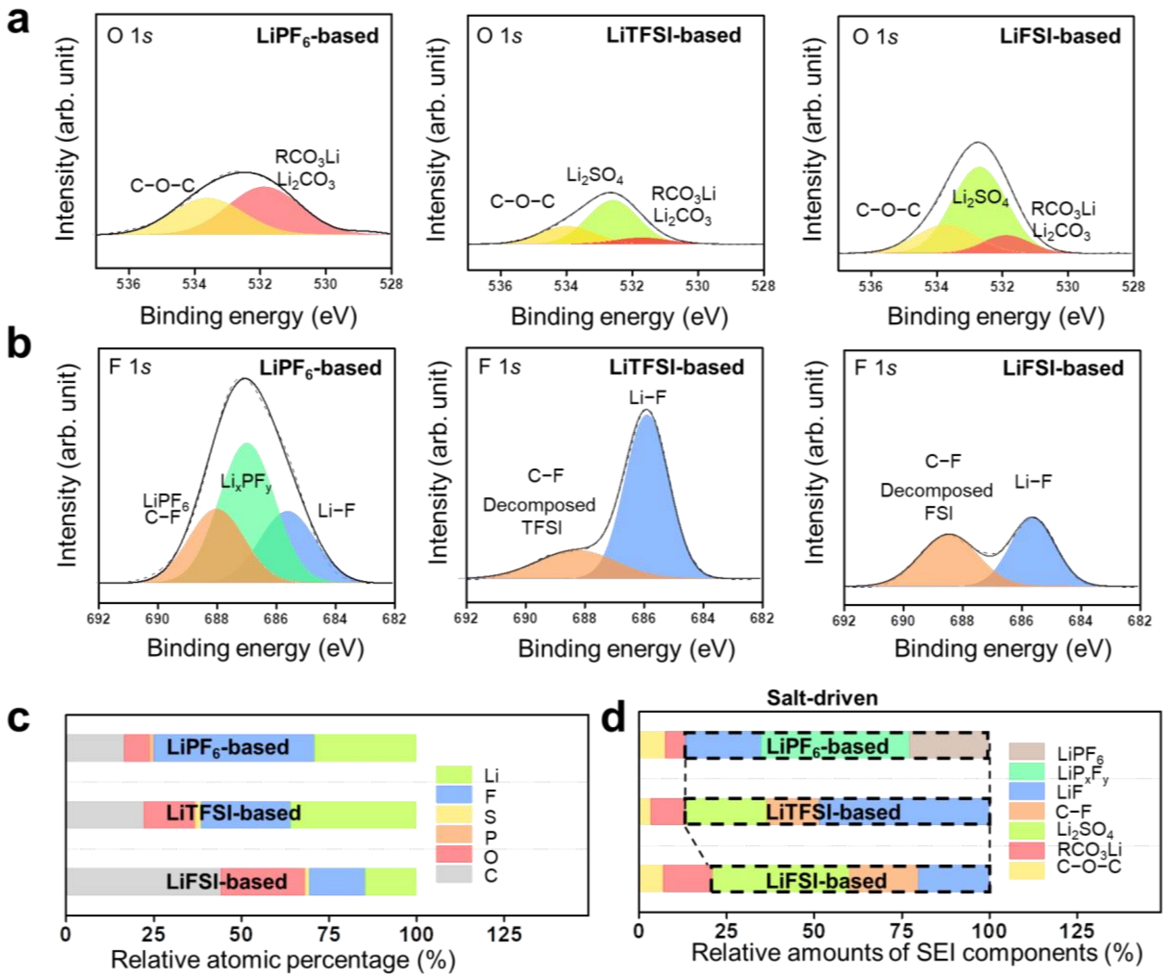

Supplementary Figure 4 | Composition analyses of photo-SEIs formed in $LiPF_6$-, LiTFSI-, and LiFSI-based electrolytes using XPS. (**a**) O 1s and (**b**) F 1s branches. (**c**) The relative atomic contents and (**d**) the relative amounts of individual components in the three photo-SEIs.

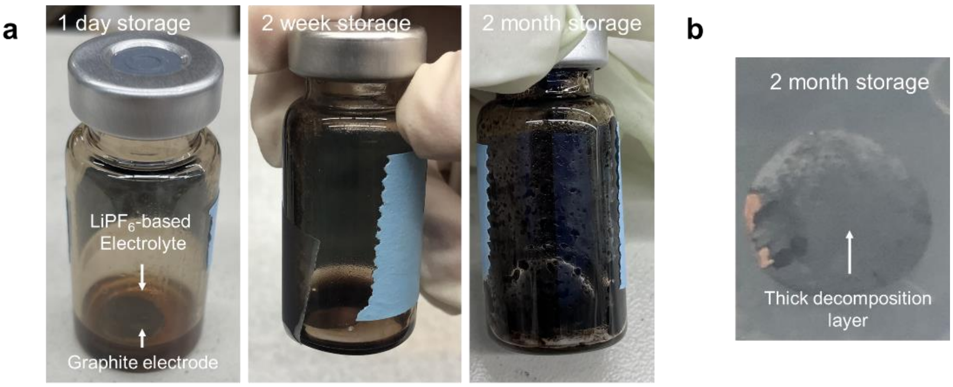

Supplementary Figure 5 | a, Photographs of the $LiPF_6$-based electrolyte stored for different periods of time after γ-ray irradiation. **b**, Photograph of graphite electrode that was kept for 2 months in the $LiPF_6$-based electrolyte upon γ-ray irradiation. A thick decomposition layer is observed.

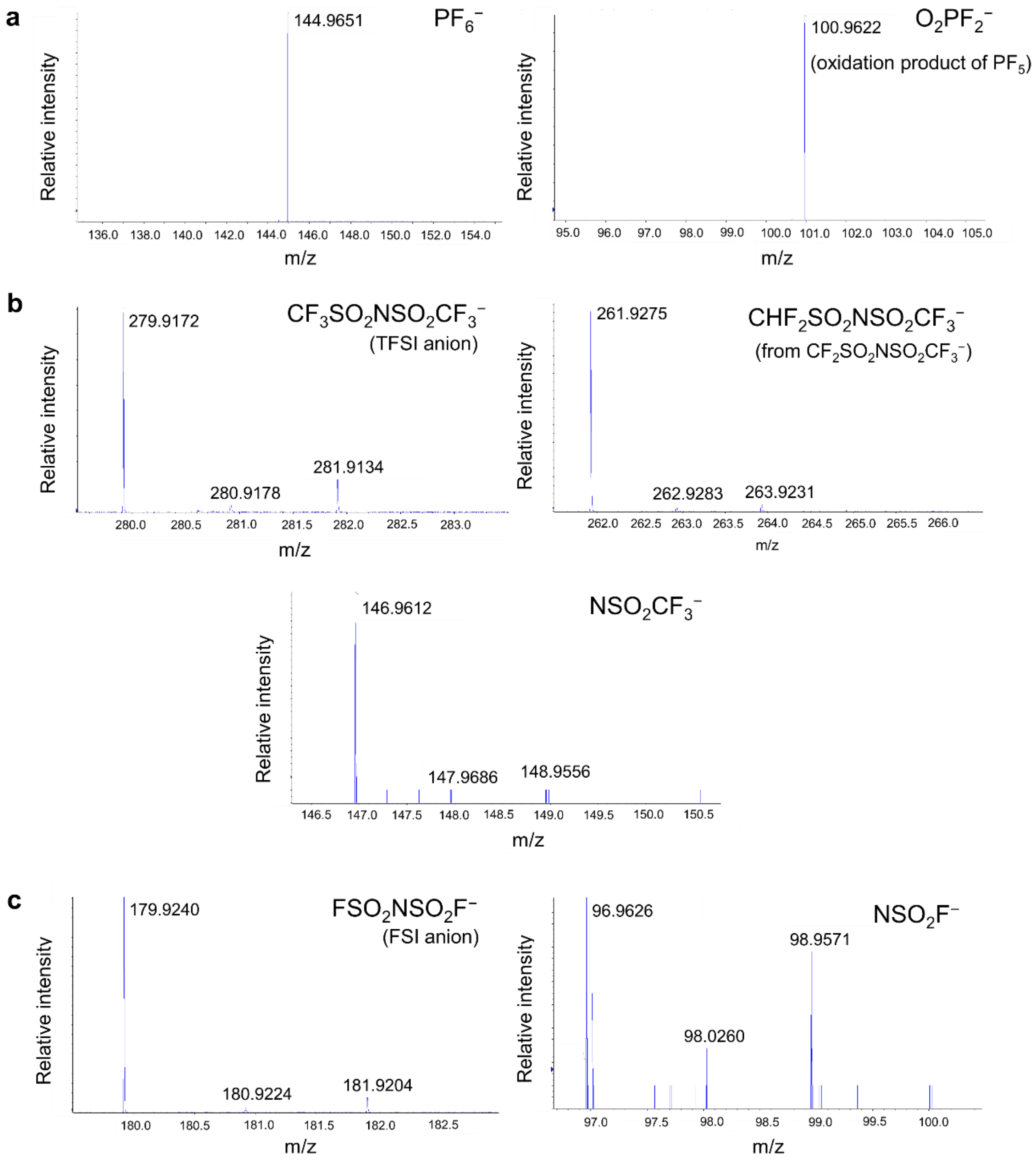

Supplementary Figure 6 | LC/MS spectra of reaction intermediates of electrolytes containing $LiPF_6$ (**a**), LiTFSI (**b**), and LiFSI (**c**) salts after γ-ray irradiation. The samples were first diluted in acetonitrile, and then chemical species were separated using an LC column and detected by the electrospray ionization (ESI) method in negative ionization mode.

$$PF_6^- + h^{+\bullet}\text{ (from graphite)} \rightarrow PF_5 + F^\bullet\text{ (1)}$$

$$F^\bullet \rightarrow h^{+\bullet}\text{ (to graphite)} + F^-\text{ (forms LiF) (2)}$$

$$N(SO_2CF_3)_2^- + e^-\text{ (from graphite)} \rightarrow (SO_2CF_3)N(SO_2CF_2)^- + F^-\text{ (forms LiF) (3)}$$

$$(SO_2CF_3)N(SO_2CF_2)^- \rightarrow (SO_2CF_3)N(SO_2CF_2) + e^-\text{ (to graphite) (4)}$$

$$N(SO_2F)_2^- + e^-\text{ (from graphite)} \rightarrow (SO_2F)NSO_2^- + F^-\text{ (forms LiF) (5)}$$

$$(SO_2F)NSO_2^- \rightarrow (SO_2F)N + SO_2 + e^-\text{ (to graphite) (6)}$$

Supplementary Figure 7 | The proposed decomposition mechanism of each salt based on the detected intermediates and the estimated dissociation energies. Equations (1–2), (3–4), and (5–6) correspond to electrolytes containing $LiPF_6$, LiTFSI, and LiFSI, respectively.

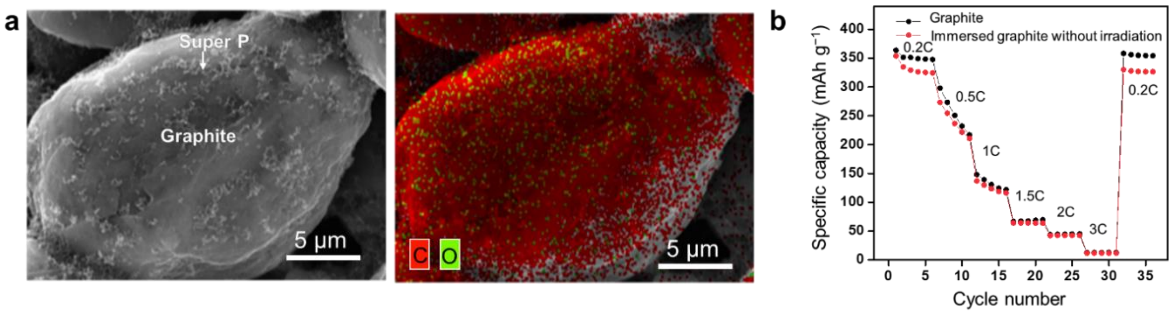

Supplementary Figure 8 | a, SEM image (left) and EDS elemental analysis (right) of a graphite electrode immersed in the electrolyte (1 M LiTFSI in EC/DEC (50/50=v/v)) for one day without γ-ray irradiation. **b**, Half-cell rate capability of the bare graphite electrode and the graphite electrode immersed in the electrolyte for one day without γ-ray irradiation.

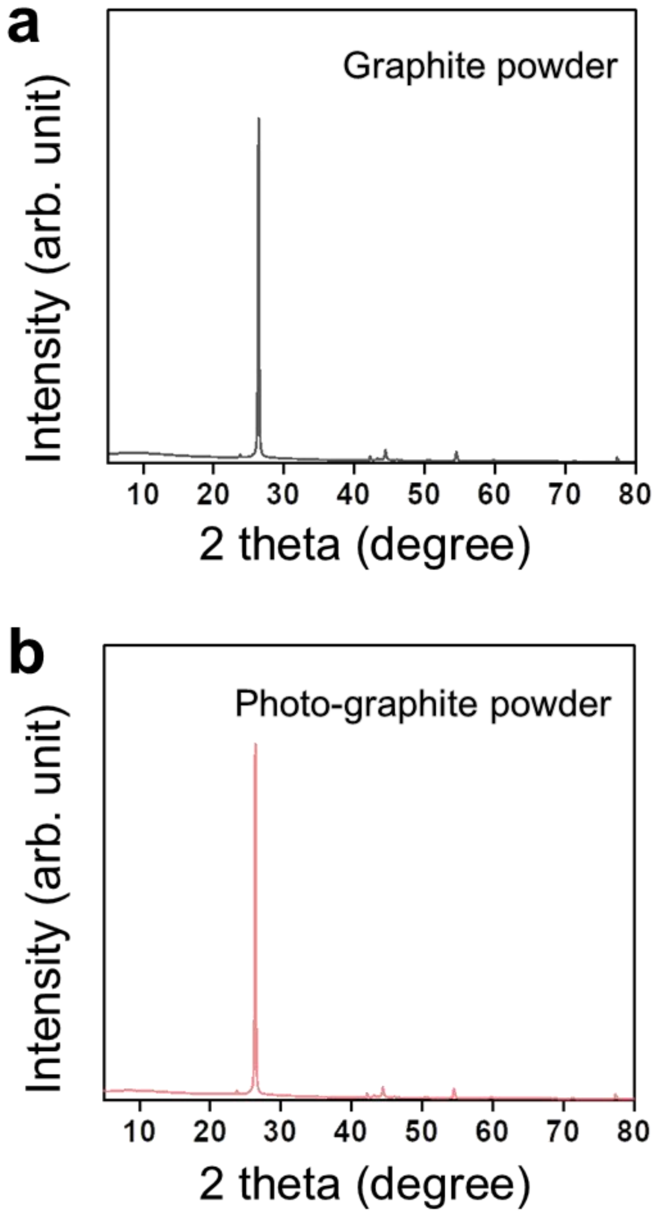

Supplementary Figure 9 | XRD patterns of (**a**) the bare graphite powder and (**b**) photo-graphite powder.

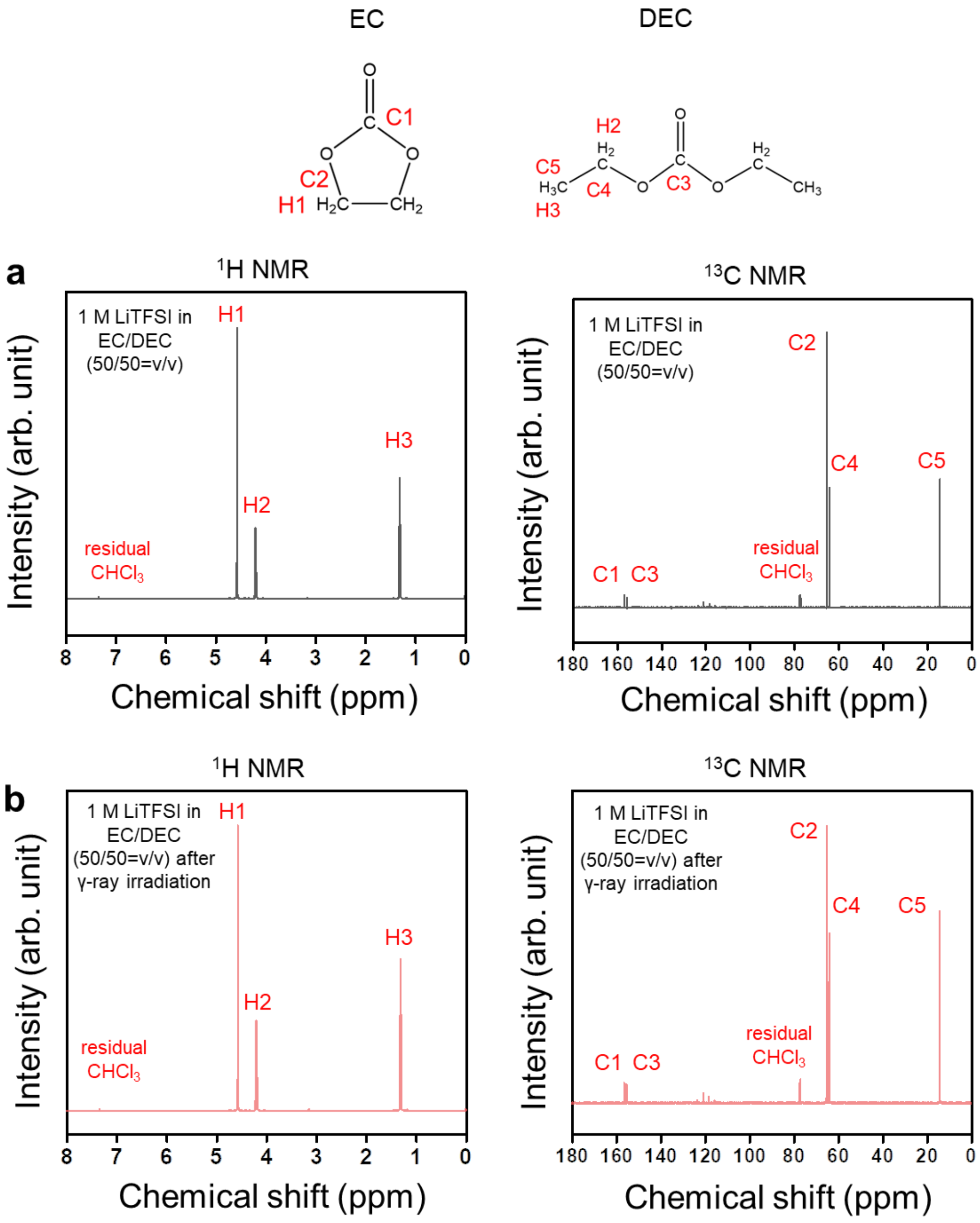

Supplementary Figure 10 | ^{1}H- and ^{13}C-NMR spectra of (**a**) 1 M LiTFSI in EC/DEC (50/50=v/v) and (**b**) 1 M LiTFSI in EC/DEC (50/50=v/v) after γ-ray irradiation. $CDCl_3$ was used as the solvent for NMR measurements.

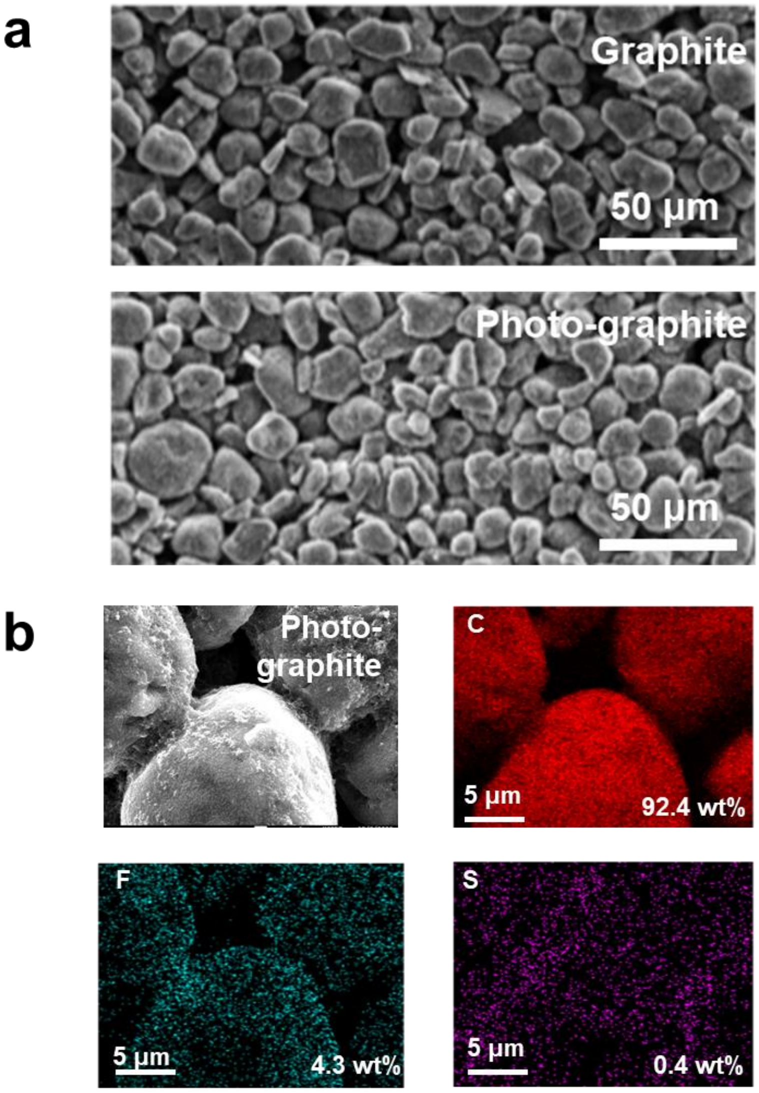

Supplementary Figure 11 | a, SEM images of the bare graphite and photo-graphite electrodes. **b**, EDS mapping results of the photo-graphite electrode.

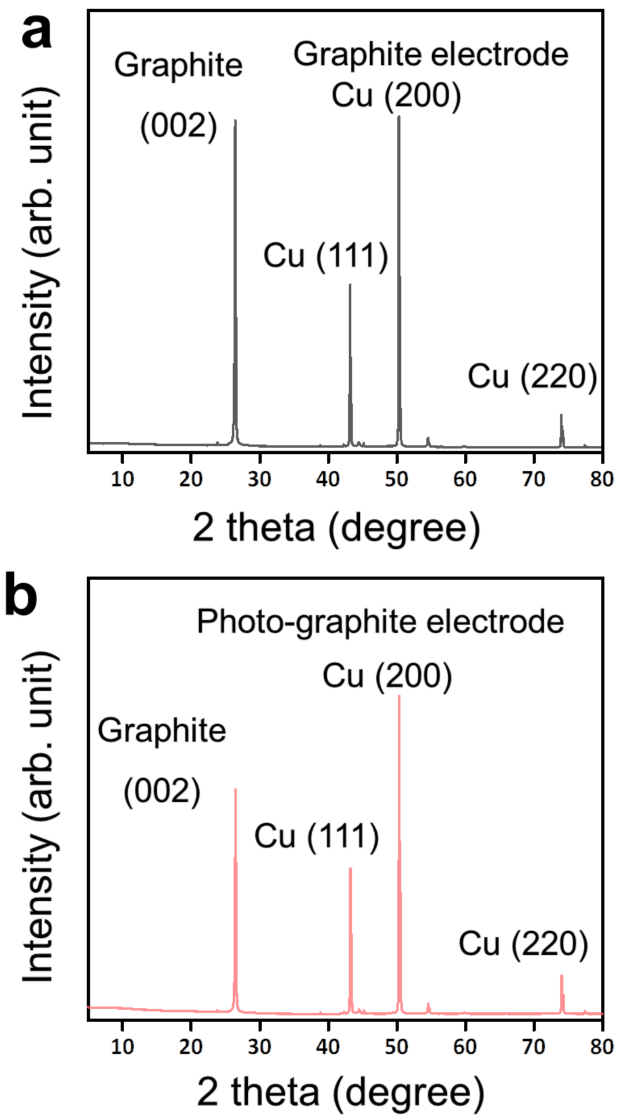

Supplementary Figure 12 | XRD patterns of (**a**) the bare graphite and (**b**) the photo-graphite electrodes.

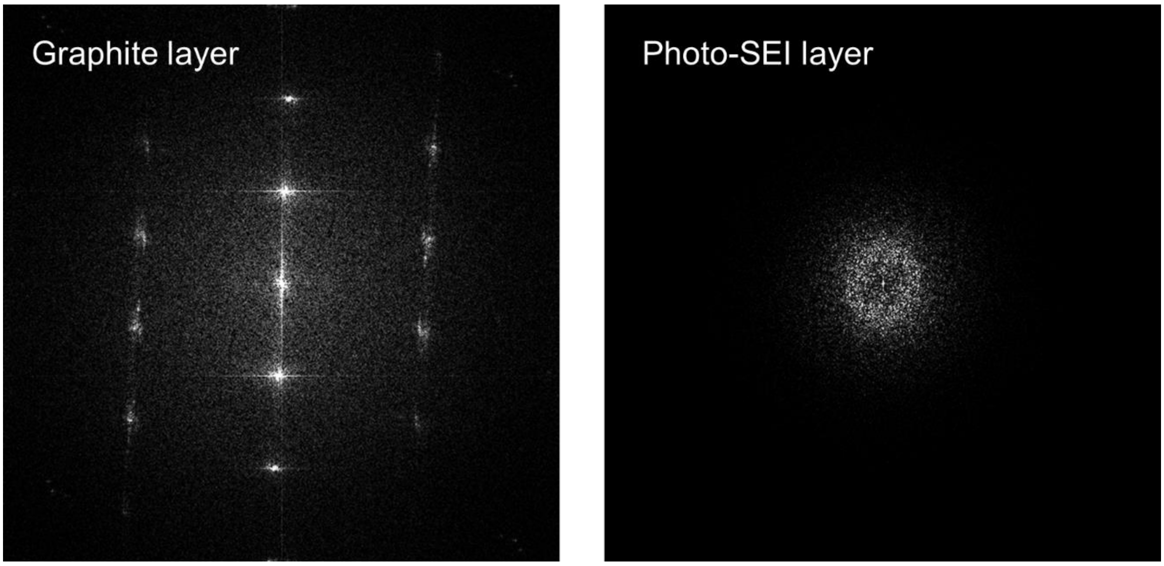

Supplementary Figure 13 | SAED patterns of the photo-graphite: bulk graphite part (left) and photo-SEI part (right).

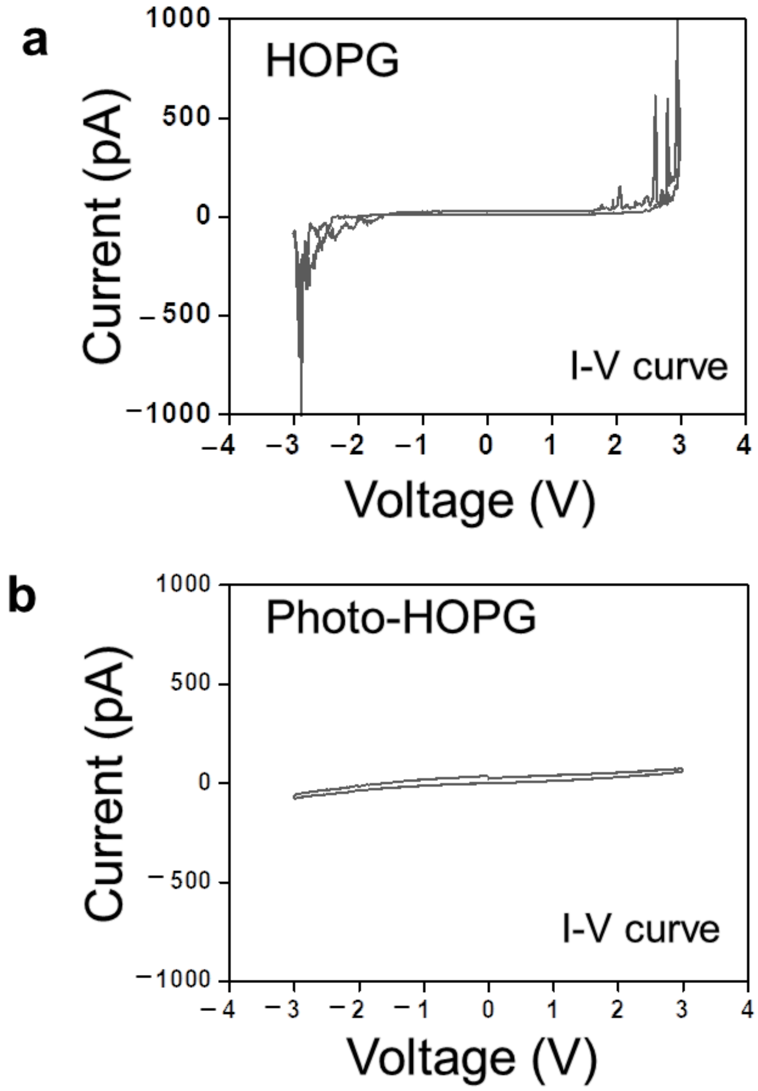

Supplementary Figure 14 | I-V curves of (**a**) the bare HOPG and (**b**) photo-HOPG using conductive AFM.

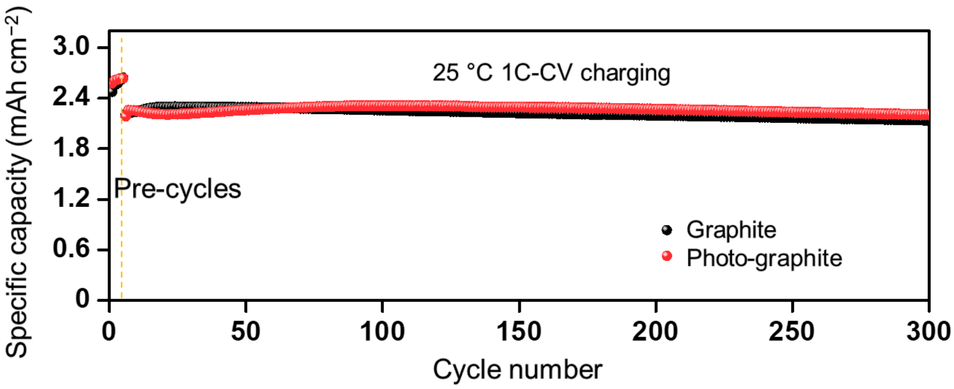

Supplementary Figure 15 | Long-term cycling stability of the bare graphite and photo-graphite full-cells in 1C-CV charging mode.

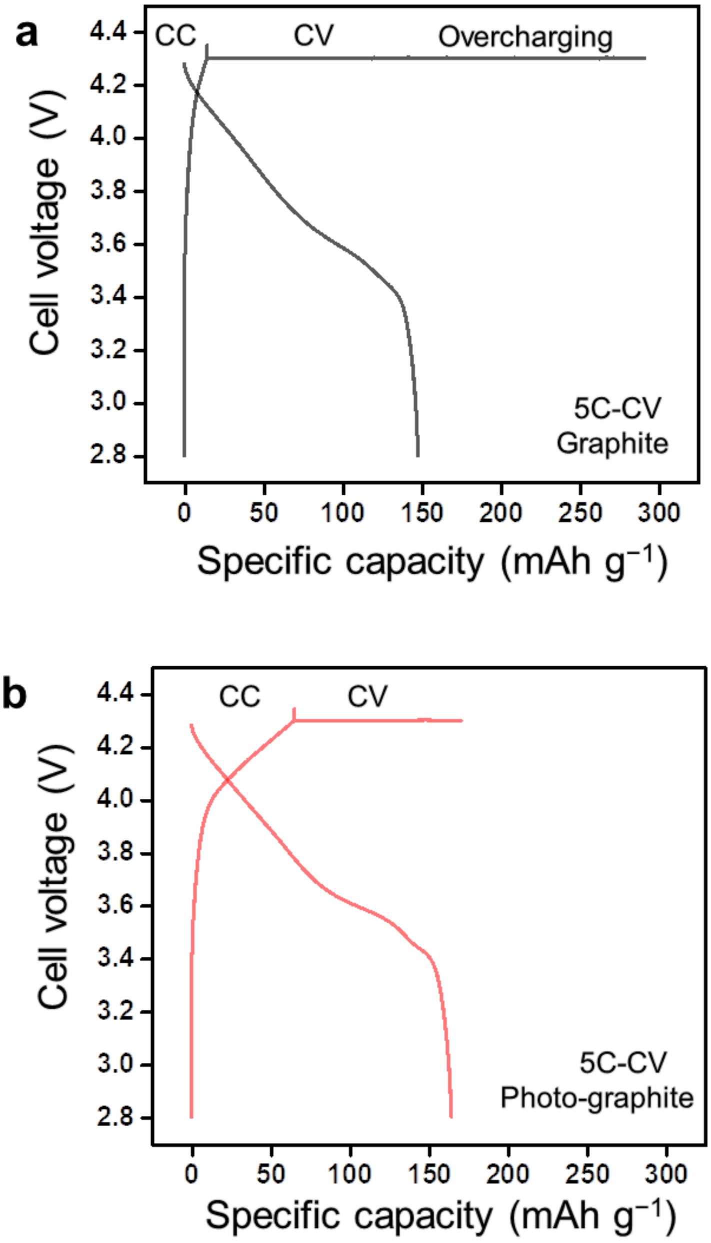

Supplementary Figure 16 | Charging and discharging voltage profiles of (**a**) the bare graphite and (**b**) the photo-graphite full-cells in 5C-CV charging mode.

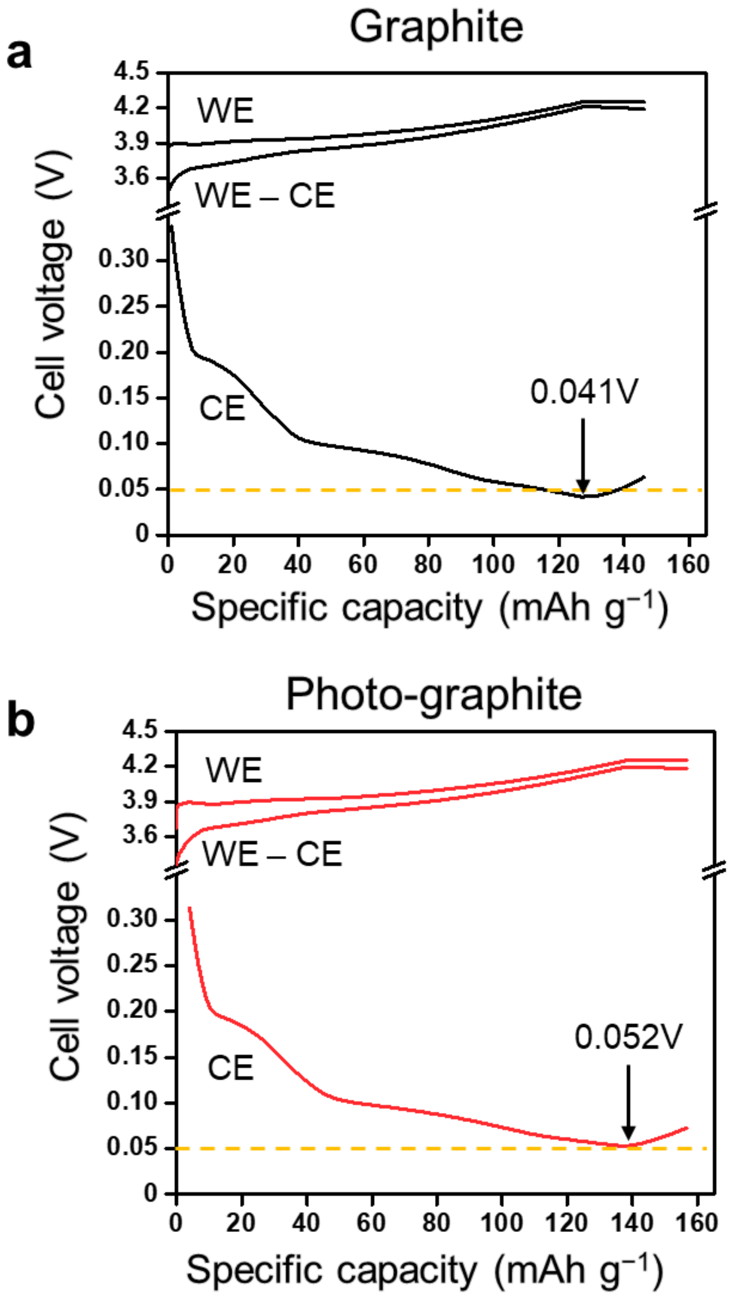

Supplementary Figure 17 | a, Voltage profiles of NCM622 cathode (WE), bare graphite anode (CE), and cell voltage (WE−CE) at 1C using a 3-electrode Swagelok cell. **b**, Voltage profiles of NCM622 cathode (WE), photo-graphite anode (CE), and cell voltage (WE−CE) at 1C using a 3-electrode Swagelok cell. For the three-electrode operation, the charging cut-off was such that the potential of the WE reached 4.25 V with respect to that of RE.

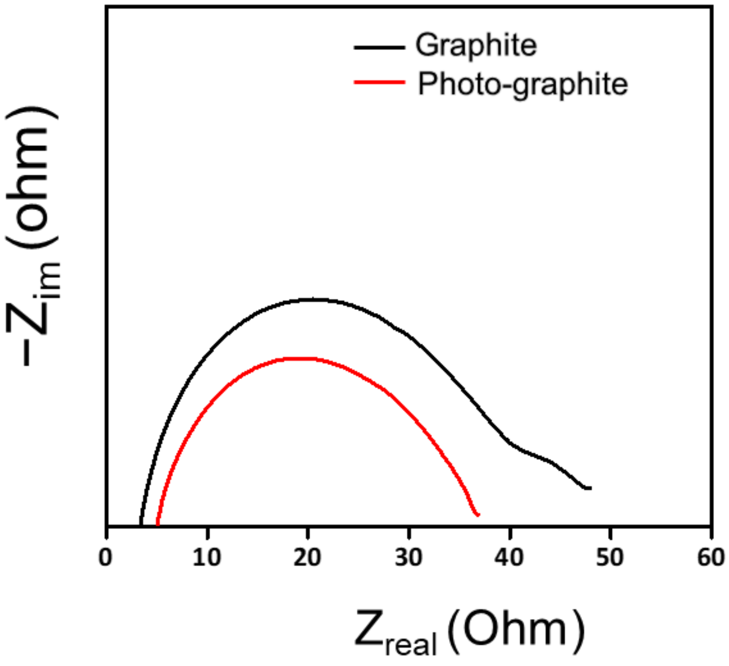

Supplementary Figure 18 | EIS profiles of the bare graphite and photo-graphite full-cells after 50 cycles in 3.5C-CV charging mode.

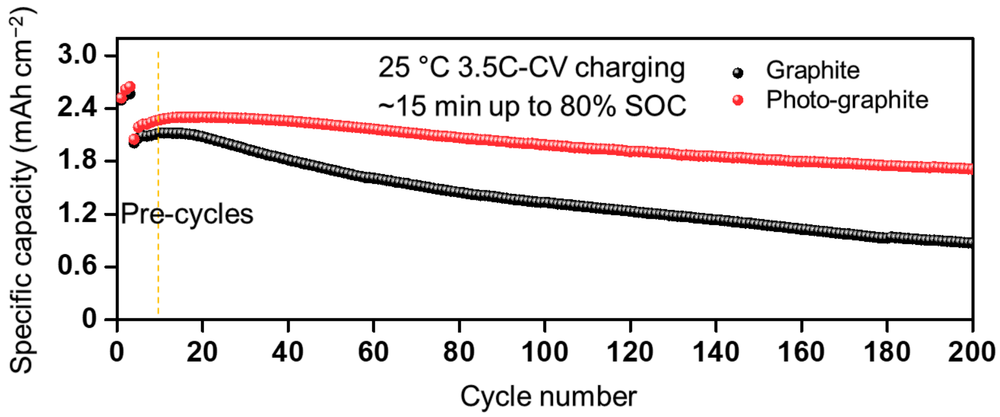

Supplementary Figure 19 | Long-term cycling performance of the bare graphite and photo-graphite full-cells at 3.5C in CC-CV charging mode.

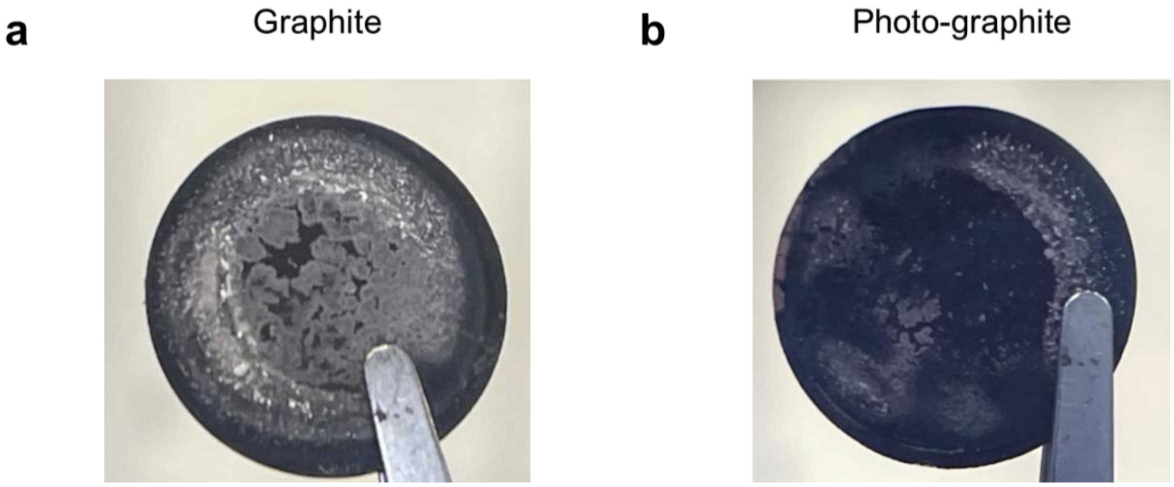

Supplementary Figure 20 | Photographic images of (**a**) the bare graphite electrode and (**b**) the photo-graphite electrode after 100 cycles in 3.5C-CV charging mode.

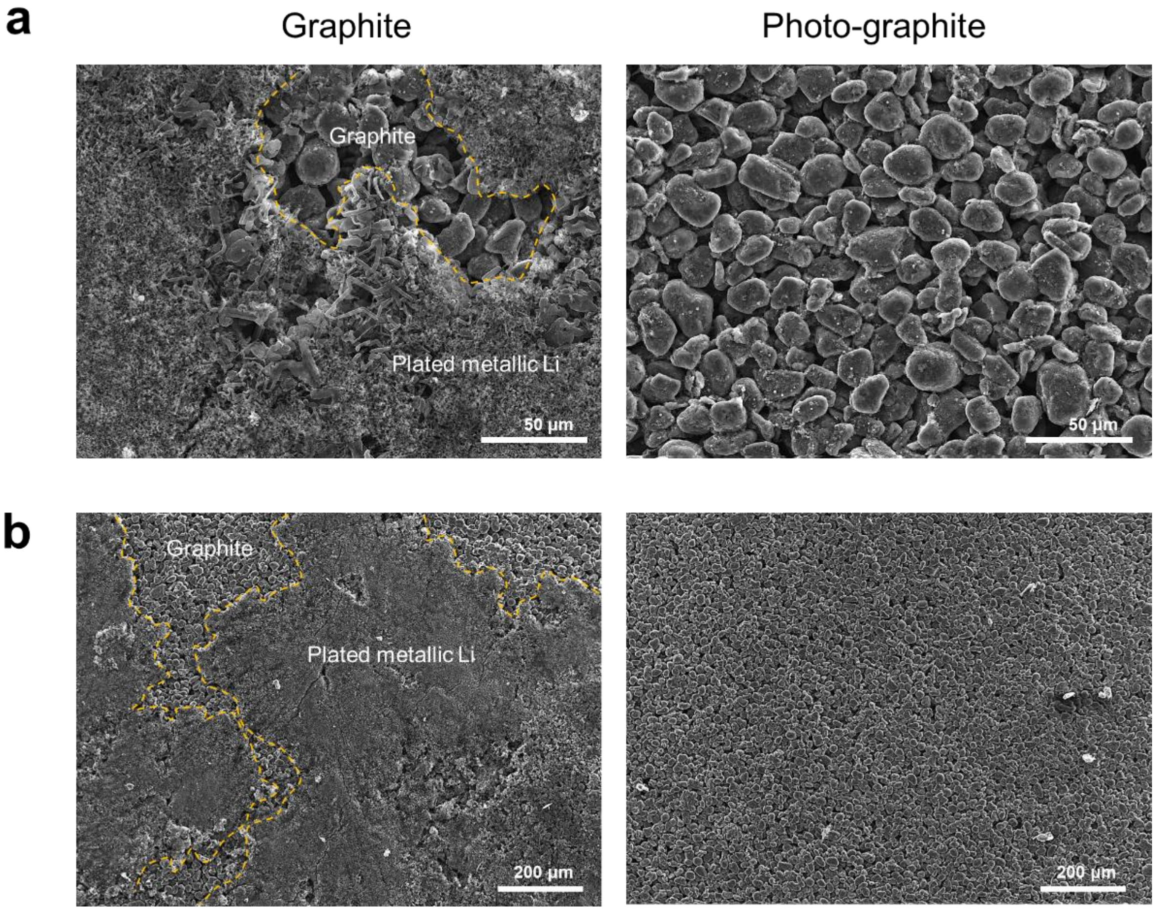

Supplementary Figure 21 | a, High-magnification SEM images of (left) the bare graphite electrode and (right) the photo-graphite electrode after 100 cycles in 3.5C-CV charging mode. **b**, Low-magnification SEM images of (left) the bare graphite electrode and (right) the photo-graphite electrode after the same 100 cycles as in **a**.

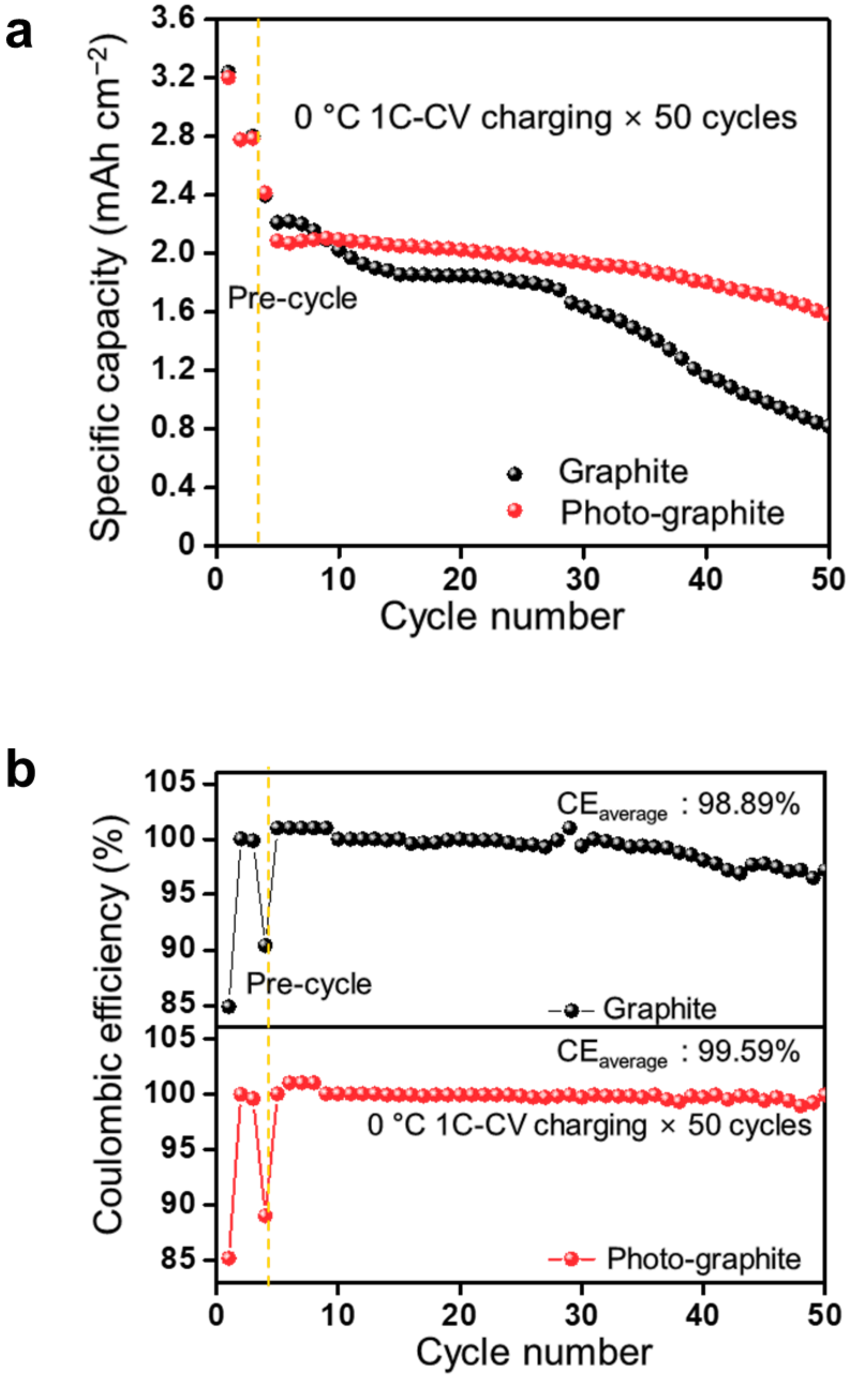

Supplementary Figure 22 | a, Cycling performance when operated in 1C-CV charging mode at 0 °C. **b**, Comparison of Coulombic efficiency when operated in 1C-CV charging mode at 0 °C.

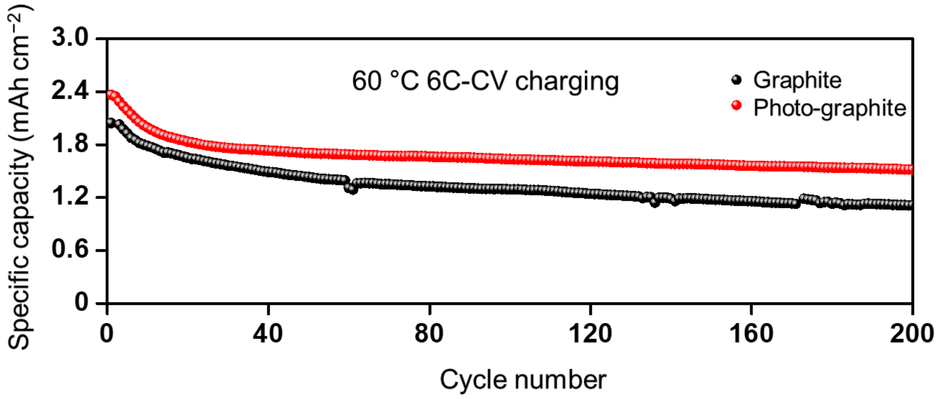

Supplementary Figure 23 | Long-term cycling performance of the bare graphite and photo-graphite full-cells at 6C with CC-CV charging mode at 60 °C.

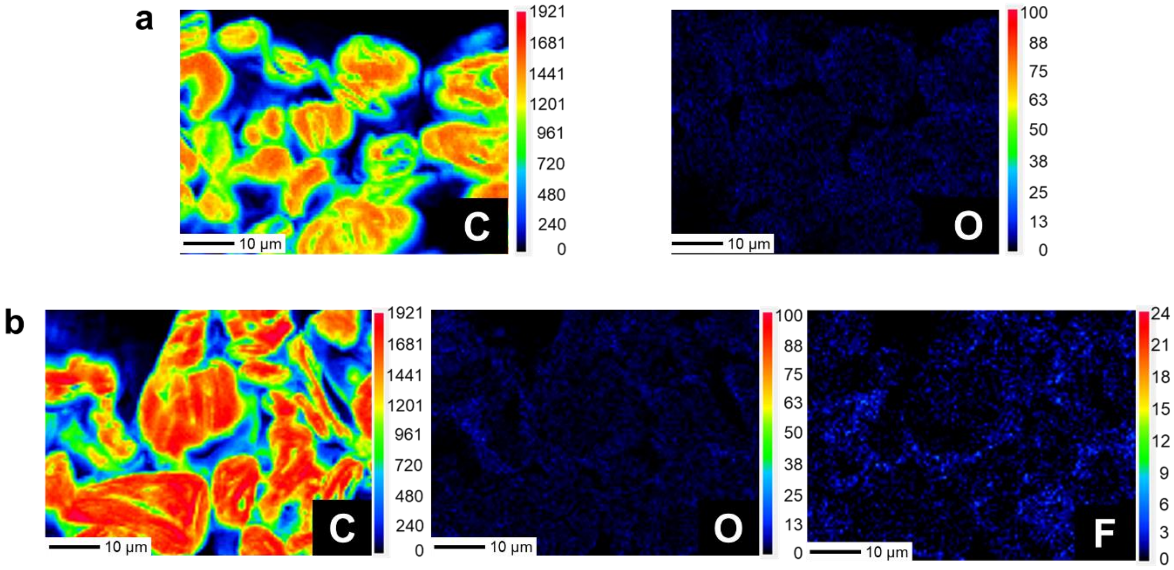

Supplementary Figure 24 | Cross-sectional FE-EPMA mapping images of (**a**) the bare graphite electrode and (**b**) the photo-graphite electrode before the rate capability tests.

Supplementary Table

	ICE[a]	Electrode composition	Areal Capacity	Fast charge capability[c]	Approach	Ref.
KOH etched graphite	92.4%	96 : 1 : 3	1.7 mAh cm^{-2}	**36.7%**	Chemical modification	S1
VGCF/carbon nanohorn graphite composite	84%	95 : 0 : 5	2−2.3 mAh cm^{-2}	**34.3%**	Heat treatment + CVD[b]	S2
Magnetically aligned graphite flakes	80%	80 : 10 : 10	1.5 mAh cm^{-2}<br>3.3 mAh cm^{-2}	**42.1%**<br>**21.5%**	Magnetic alignment	S3
Silicon nanolayer in Edge-plane activated graphite	93.8%	96 : 1 : 3<br>(7 wt% silicon)	3.3 mAh cm^{-2}	**68%**	CVD[b]	S4
Photochemical SEI graphite	94.3%	93 : 3 : 4	2.9 mAh cm^{-2}	**70.5%**	Photochemical	This work

a. Initial Coulombic Efficiency (ICE)

b. Chemical Vapor Deposition (CVD)

c. Lithiation capacity retention at 1C in half-cell

Supplementary Table 1 | Fast charging performance of graphite anodes in previous reports.

Supplementary References

[1] Cheng, Q., Yuge, R., Nakahara, K., Tamura, N. & Miyamoto, S. KOH etched graphite for fast chargeable lithium-ion batteries. *J. Power Sources* **284**, 258−263 (2015).

[2] Yuge, R., Tamura, N., Manako, T., Nakano, K. & Nakahara, K. High-rate charge/discharge properties of Li-ion battery using carbon-coated composites of graphites, vapor grown carbon fibers, and carbon nanohorns. *J. Power Sources* **266**, 471−474 (2014).

[3] Billaud, J., Bouville, F., Magrini, T., Villevieille, C. & Studart, A. R. Magnetically aligned graphite electrodes for high-rate performance Li-ion batteries. *Nat. Energy* **1,** 16097 (2016).

[4] Kim, N., Chae, S., Ma, J., Ko, M. & Cho, J. Fast-charging high-energy lithium-ion batteries via implantation of amorphous silicon nanolayer in edge-plane activated graphite anodes. *Nat. Commun.* **8**, 812 (2017).
